# Rh(III)-catalyzed chelation-assisted C–H activation/annulation of 2-arylimidazolines with cyclic diazo-1,3-dicarbonyl compounds: a novel approach to tetracyclic annulated derivatives of 2,3-dihydroimidazo[2,1-*a*]isoquinoline

**DOI:** 10.3762/bjoc.22.78

**Published:** 2026-06-30

**Authors:** Ivan Lyutin, Grigory Kantin, Olga Bakulina, Dmitry Dar'in

**Affiliations:** 1 Institute of Chemistry, Saint Petersburg State University, 26 Universitetskyi prospekt, Peterhof 198504, Russian Federation

**Keywords:** annulation, 2-arylimidazolines, catalysis, С–H activation, diazodiketones, 2,3-dihydroimidazo[2,1-*a*]isoquinolines, dimedone, tetraheterocycles

## Abstract

2-Arylimidazolines were annulated with cyclic diazo-1,3-dicarbonyl compounds under Rh(III)-catalysis for the first time. The developed general and efficient approach based on CH-activation allowed the efficient preparation of five novel types of tetraheterocyclic systems derived from 2,3-dihydroimidazo[2,1-*a*]isoquinolines fused with an additional five-, six- or seven-membered carbocycle or *N*-/*O*-heterocycle.

## Introduction

**Figure 1:**
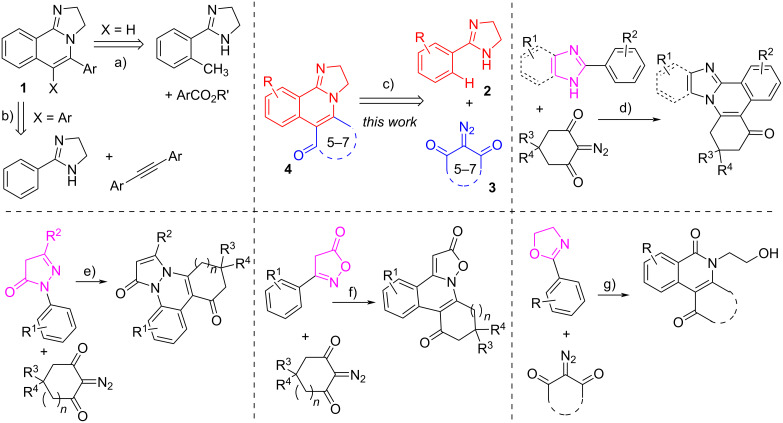
a,b) Structure of biologically active 2,3-dihydroimidazo[2,1-*a*]isoquinolines (1) and their published syntheses. c) A novel approach to annulated 2,3-dihydroimidazo[2,1-*a*]isoquinolines via chelation-assisted C–H activation/annulation of 2-arylimidazolines proposed in this work. d–g): Published examples of chelation-assisted C–H activation/annulation of other five-membered heterocycles.

The advancement of organic synthesis techniques, especially methods for creating nitrogen-containing heterocycles, is essential for the creation of more potent medications and the hunt for novel biologically active compounds. One important group of these are imidazoline derivatives since they are well known as pharmacophores [1] for adrenergic and imidazoline receptors. Imidazolines have found utility in the development of many drug candidates and marketed drugs for the treatment of hypertension [2], diabetes [3], and various central nervous system disorders [4]. Recently, imidazolines that exhibit anticancer activity, like nutlins [5–6], have also garnered a lot of interest. There are numerous general methods for synthesizing imidazolines [7–8], but access to their annulated derivatives is still very limited despite many promising results on their bioactivity. For example, several derivatives of 5-aryl-2,3-dihydroimidazo[2,1-*a*]isoquinolines (Figure 1, **1**) have demonstrated antitumor [9–11], anti-inflammatory, and immunosuppressive activity. The latter was used to develop antiasthmatic, pulmonary, and anti-allergy agents acting as platelet-activating factor receptor (PAF-R) antagonists [12–13]. Derivatives with X = H were accessed only via lithiation of 2-(*o*-tolyl)imidazolines followed by condensation with aromatic esters (Figure 1a) [12,14–15], while analogs with an additional aryl group at position 6 were obtained only once as part of investigation of annulation of arylamidines with internal alkynes under ruthenium catalysis (Figure 1b) [16]. Regarding this, we were interested in developing a general approach to obtaining structurally related 5,6-disubstituted 2,3-dihydroimidazo[2,1-*a*]isoquinolines **4** (Figure 1c) fused with an additional ring via a Rh(III)-catalyzed chelation-assisted C–H activation/annulation with diazo compounds. 2-Arylimidazolines **2** and various substituted cyclic 1,3-dicarbonyl diazo compounds **3** with ring sizes from 5 to 7 atoms were chosen as key building blocks. This strategy [17] has already proven to be a powerful tool for the synthesis of diverse fused polycyclic compounds using functionalized benzenes or six-membered aryl-substituted heterocycles as substrates, but there were only a few published examples of employing aryl derivatives of five-membered heterocycles. Most of these publications describe annulation of imidazoles or benzimidazoles (Figure 1d) [18–20]. Additional rare examples include pyrazolones (Figure 1e) [21], isoxazolones (Figure 1f) [22], and oxazolines (Figure 1g) [23]. Interestingly, the latter reacted with ring-opening of the initial heterocyclic moiety. In the present work, we have extended this strategy to annulation of 2-arylimidazolines, which opened the way to several types of new medicinally relevant azatetraheterocycles having a 2,3-dihydroimidazo[2,1-*a*]isoquinoline core.

## Results and Discussion

All necessary substrates for the developed approach were easily accessed via standard procedures: diazo compounds **3** were obtained [24] using the Regitz diazo transfer for corresponding commercially available 1,3-dicarbonyl compounds with arylsulfonyl azides, while 2-arylimidazolines **2** were prepared via condensation of aromatic aldehydes with ethylenediamine in the presence of *N*-bromosuccinimide [25]. We began our investigation with the model reaction of 2-(4-chlorophenyl)imidazoline (**2a**) with 2-diazodimedone (**3a**), which resulted in the formation of 2,5,6,7-tetrahydroimidazo[1,2-*f*]phenanthridin-8(3*H*)-one **4a** (Table 1). The highest isolated yield of 88% was achieved for a reaction conducted in trifluoroethanol (TFE) at 80 °C for 12 h using 2.5 mol % of [RhCp*Cl_2_]_2_, (where Cp* = pentamethylcyclopentediene) and 10 mol % of silver triflimide as a catalytic system (Table 1, entry 1). A screening of the reaction conditions revealed that the formation of **4a** can be promoted by several other additives such as sodium acetate, acetic acid, silver hexafluoroantimonate, or silver triflate, but all of them provided much lower yields (Table 1, entries 2–5). Reducing the amount of silver triflimide also proved less effective (Table 1, entry 6), and, without the additive, the target product was observed in trace amounts insufficient for isolation (Table 1, entry 7). This suggests that the [RhCp*Cl_2_]_2_ complex cannot catalyze the reaction itself. Shortening the reaction time or changing the temperature did not improve the result as well (Table 1, entries 8–11). The type of solvent was also found to be critical for this reaction – using dichloroethane allowed preparation of compound **4a** in a lower, but still noticeable, yield of 60%, while no target product was isolated from acetonitrile (Table 1, entries 12–13).

**Table 1:** Screening of reaction conditions^a^. Synthesis of compound **4a**.

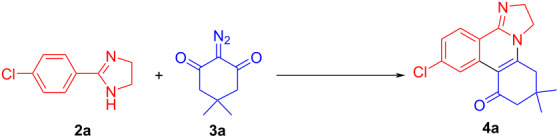					

Entry	Solvent	Additive (mol %)	Temperature (°C)	Time (h)	Yield^b^ (%)

**1**	**TFE**	**AgNTf** **_2 _** **(10)**	**80**	**12**	**88**
2	TFE	NaOAc (20)	80	12	36
3	TFE	HOAc (200)	80	12	43
4	TFE	AgSbF_6_ (10)	80	12	26
5	TFE	AgOTf (10)	80	12	65
6	TFE	AgNTf_2_ (5)	80	12	68
7	TFE	–	80	12	traces*^c^*
8	TFE	AgNTf_2_ (10)	80	2	46
9	TFE	AgNTf_2_ (10)	80	6	67
10	TFE	AgNTf_2_ (10)	70	12	80
11	TFE	AgNTf_2_ (10)	90	12	85
12	DCE	AgNTf_2_ (10)	80	12	60
13	MeCN	AgNTf_2_ (10)	80	12	traces*^c^*

^a^Reaction conditions: 0.24 mmol of diazo compound **3a**, 0.2 mmol of imidazoline **2a**, 2 mL of solvent, 2.5 mol % of [RhCp*Cl_2_]_2_. ^b^Isolated yield. TFE = 2,2,2-triflouroethanol, DCE = 1,2-dichloroethane, Tf = trifluoromethanesulfonyl. ^c^The product was detected in the NMR spectrum, but was not isolated due to low content.

**Scheme 1:**
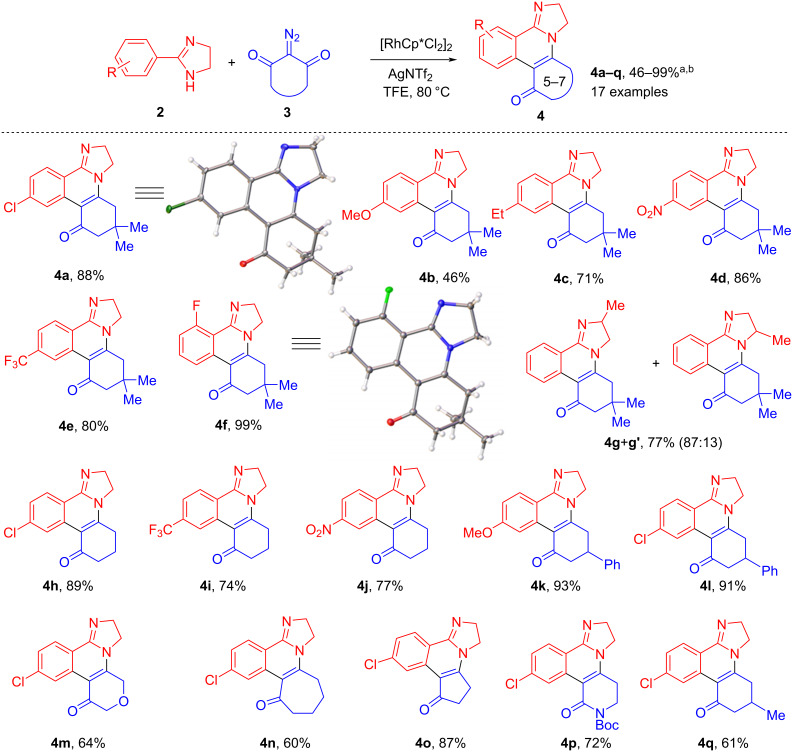
C–H activation/annulation of 2-arylimidazolines with diazo-1,3-dicarbonyl compounds. ^a^Reaction conditions: imidazoline **2** (0.2 mmol), diazo compound **3** (0.24 mmol, 1.2 equiv), [RhCp*Cl_2_]_2_ (2.5 mol %) and AgNTf_2_ (10 mol %), TFE (2 mL), 80 °C, 12 h. ^b^Isolated yields.

In order to investigate the scope and limitations of the developed approach, we proceeded with the substrate variation (Scheme 1). First, the aryl component of imidazoline was altered. All tested aryls – phenyl and EWG (Cl, NO_2_, CF_3_) or EDG (OMe, Et)-substituted benzenes were well tolerated, including *ortho*-substituted derivatives. When imidazolines with heteroaromatic groups (4-pyridine and 2-furane, compounds **2h** and **2i**, Supporting Information File 1) at position 2 were used, only traces of corresponding products were observed. This was not unexpected, as we were unable to find any published successful examples of Rh(III)-catalyzed chelation-assisted C–H activation/annulation with diazo compounds when such heterocyclic substrates were used as CH-components. Substitution at position 4 of the imidazoline ring with a methyl group resulted in the formation of an inseparable regioisomeric mixture of compounds **4g** and **4g'** (ratio = 87:13) due to imidazoline tautomerization. Less sterically hindered isomer **4g** was formed predominantly, which was supported by the NOESY spectrum (Supporting Information File 1). An interaction between protons from the methylene group of the dimedone moiety at 4.18 ppm with both protons from the methylene group of the imidazoline moiety at 2.55 and 3.60 ppm was observed for the major isomer **4g**.

Next, the scope of suitable diazo carbonyl compounds was examined. In addition to dimedone, 1,3-cyclohexanedione and its 5-phenyl and 5-methyl-substituted derivatives provided a series of products **4h–l** and **4q** in 61–93% yields. To our delight, 5-oxa- and 4-aza-analogs of diazo-1,3-cyclohexanedione and diazo 1,3-diketones with other ring sizes (5 and 7) were also found suitable for this reaction, which allowed preparation of four more types of tetracyclic imidazoline-based systems, represented by compounds **4m**, **4p**, **4o**, **4n**. Isolation of compounds **4m** and **4p** is a rare example of involving derivatives of 2-diazocyclohexane-1,3-dione with additional endocyclic heteroatoms (compounds **3g** and **3h**) in Rh(III)-catalyzed chelation-assisted C–H activation/annulation. The structures of compounds **4a** and **4f** were supported by X-ray crystallography (CCDC 2450071 and 2502221).

**Figure 2:**
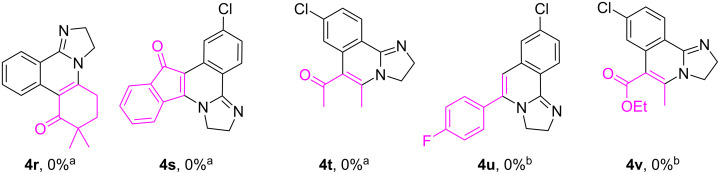
Structures of products from unsuccessful reactions of 2-arylimidazolines with diazo 1,3-dicarbonyl compounds. ^a^Trace amounts were detected by ^1^H NMR of crude reaction mixtures. ^b^Not observed by ^1^H NMR of crude reaction mixtures.

Several other types of diazo compounds were found to be unsuitable for this approach since products **4r–v** could not be isolated (Figure 2). These diazo compounds were derived from α,α-disubstituted 1,3-cyclohexanedione, benzoannulated five-membered diketone, acyclic diketone, acetophenone, and acyclic keto ester. In the case of compounds **4u** and **4v**, no desired reaction was observed, while for compounds **4r–t**, despite some small amount of the product was detected in crude reaction mixtures by NMR, none of them could be isolated in pure form by column chromatography.

**Figure 3:**
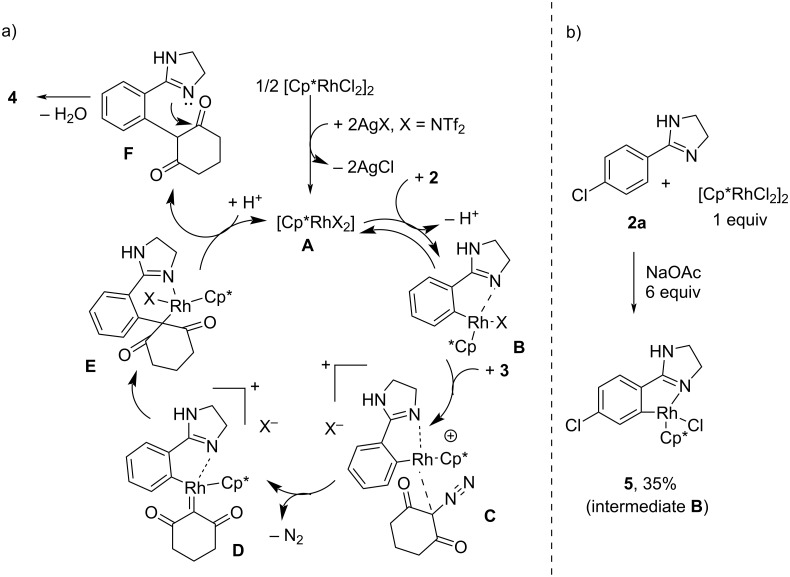
a) Proposed reaction mechanism; b) Experimental isolation of intermediate **B** (compound **5**) from equimolar reaction of substrate **2a** and the catalyst.

The following reaction mechanism can be proposed according to the literature data [18] (Figure 3a). The catalytic cycle begins with the formation of monomeric Rh(III)-catalyst **A** from the reaction of [RhCp*Cl_2_]_2_ with AgNTf_2_. An external non-bridging anion such as NTf is required to replace the bridging chloride ligand, which converts the inert dimeric rhodium complex into the reactive monomer. Next, a Rh(III)-catalyzed chelation-assisted C–H activation of imidazoline **2** occurs, generating a rhodacyclic intermediate **B**, followed by coordination of diazo compound **3** to give diazonium intermediate **C**. The latter transforms into a carbene complex **D** after nitrogen loss. The next intermediate **E** is a result of migratory insertion of the carbenoid into the C–Rh bond. The last step of the catalytic cycle is protodemetallation that delivers C_Ar_-alkylated intermediate **F** and regenerates the active catalytic species. The target tetraheterocyclic product **4** is formed after intramolecular condensation between the carbonyl group and the imidazoline nitrogen of intermediate **F**. To support this mechanism experimentally, we have performed a reaction of substrate **2a** with an equimolar amount of rhodium catalyst, which allowed isolation of compound **5** (intermediate **B**) in 35% yield (Figure 3b).

## Conclusion

In conclusion, we have successfully extended the strategy of Rh(III)-catalyzed chelation-assisted C–H activation/annulation with diazo compounds to 2-arylimidazolines. This general approach allowed the simple and efficient assembly of five novel types of tetraheterocyclic systems derived from 2,3-dihydroimidazo[2,1-*a*]isoquinolines fused with an additional five-, six-, or seven-membered cycle from readily available starting materials. The scope and limitations of this annulation were thoroughly investigated. Five types of cyclic diazo 1,3-dicarbonyl compounds (derived from dimedone, 5- or 6-substituted cyclohexanediones, cyclopentanedione, and cycloheptanedione) and two types of 2-arylimidazolines (with or without additional substituent at the imidazoline ring) were successfully introduced into the target reaction. Two heterocyclic diazo diketones were introduced to this annulation approach for the first time. This strategy allowed preparation of 17 novel structurally diverse annulated derivatives of 2,3-dihydroimidazo[2,1-*a*]isoquinoline with variation of substituents at four different positions. The structural similarity of the prepared compounds with known 2,3-dihydroimidazo[2,1-*a*]isoquinoline-based antitumor, anti-inflammatory, and immunosuppressive agents makes the developed approach a useful tool for the development of novel biologically active compounds.

## Supporting Information

File 1 Detailed experimental procedures for the preparation of compounds **2–4**, analytical data for compounds **4a–q**, copies of their NMR spectra, and X-ray crystallography data for compounds **4a** and **4f**.

## Data Availability

Data generated and analyzed during this study is available from the corresponding author upon reasonable request.
