# Supplementary material for: Rh(III)-catalyzed chelation-assisted C–H activation/annulation of 2-arylimidazolines with cyclic diazo-1,3-dicarbonyl compounds: a novel approach to tetracyclic annulated derivatives of 2,3-dihydroimidazo[2,1-a]isoquinoline
Source: Beilstein J Org Chem. 2026 Jun 30;22:997–1003. doi: 10.3762/bjoc.22.78 (PMC13338596; doi:10.3762/bjoc.22.78)
Supplement: File 1 — Detailed experimental procedures for the preparation of compounds 2–4, analytical data for compounds 4a–q, copies of their NMR spectra, and X-ray crystallography data for compounds 4a and 4f. [file Beilstein_J_Org_Chem-22-997-s001.pdf]

BEILSTEIN JOURNAL OF ORGANIC CHEMISTRY

Supporting Information

for

Rh(III)-catalyzed chelation-assisted C–H activation/annulation of 2-arylimidazolines with cyclic diazo-1,3-dicarbonyl compounds: a novel approach to tetracyclic annulated derivatives of 2,3-dihydroimidazo[2,1-*a*]isoquinoline

Ivan Lyutin, Grigory Kantin, Olga Bakulina and Dmitry Dar'in

Beilstein J. Org. Chem. **2026,** *22,* 997–1003. doi:10.3762/bjoc.22.78

Detailed experimental procedures for the preparation of compounds 2–4, analytical data for compounds 4a–q, copies of their NMR spectra, and X-ray crystallography data for compounds 4a and 4f

License and Terms: This is a supporting information file under the terms of the Creative Commons Attribution License (https://creativecommons.org/licenses/by/4.0). Please note that the reuse, redistribution and reproduction in particular requires that the author(s) and source are credited and that individual graphics may be subject to special legal provisions.
The license is subject to the *Beilstein Journal of Organic Chemistry* terms and conditions: (https://www.beilstein-journals.org/bjoc/terms)

Table of contents

1. General information S2
2. Experimental procedures S3
2.1 Preparation of the imidazolines **2a–i** S3
2.2 Preparation of diazo compounds **3a–m** S3
2.3 Preparation of imidazoisoquinolines **4a–q** S4
2. Copies of NMR spectra S12
3. Crystallographic data for compounds **4a** and **4f** S33
4. References S35

1. General information

Solvents: Unless otherwise indicated, solvents were obtained from commercial suppliers. Dichloromethane (DCM) was dried by distillation from P_2O_5 and stored over molecular sieves (4 Å).

Reagents: Unless otherwise indicated, reagents were used as purchased from commercial suppliers. $[RhCp^*Cl_2]_2$ was obtained from $RhCl_3$ and 1,2,3,4,5-pentamethylcyclopentadiene according to the previously published protocol[1].

Nuclear magnetic resonance spectroscopy: NMR spectroscopic data were recorded with a Bruker Avance III 400 MHz spectrometer (400.13 MHz for ^{1}H and NOESY 100.61 MHz for ^{13}C{^{1}H} and 376.50 MHz for ^{19}F{^{1}H}), and with a Bruker Avance III 500 MHz spectrometer (125.73 MHz for ^{13}C{^{1}H}) in $CDCl_3$ and DMSO-d_6 and were referenced to residual solvent proton signals (δ_H = 7.26 and 2.50 ppm, respectively) and solvent carbon signals (δ_C = 77.16 and 39.52 ppm, respectively).

Melting points: Melting points were determined with a melting point apparatus REACH Devices RD-MP in the open capillary tubes.

Mass spectrometry: HRMS were recorded using a microOTOF-Q spectrometer (Brucker); ionization by electrospray, positive detection.

X-ray crystallography: Single crystal X-ray data were obtained using an Agilent Technologies SuperNova Atlas and an Agilent Technologies Xcalibur Eos diffractometers at a temperature of 100 K.

Thin layer chromatography: Thin layer chromatography (TLC) was performed on aluminum-backed pre-coated plates with silica gel 60 F_{254} with a suitable solvent system and was visualized using UV fluorescence.

Column chromatography: Column chromatography was carried out with silica gel grade 60 (0.040−0.063 mm) 230−400 mesh using Biotage Isolera Prime instrument.

Heating: An aluminum heating block was used to maintain the temperature of the reaction mixtures.

2. Experimental procedures

2.1 Preparation of the imidazolines 2a–i

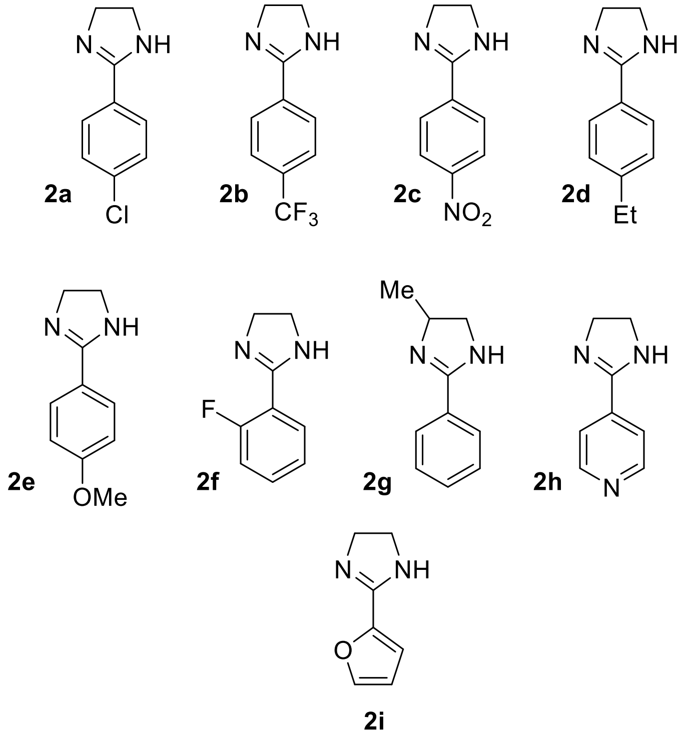

Imidazolines **2a–i** were prepared according to the common protocol for condensation of equimolar amounts of diamine and arylaldehyde in dry DCM overnight at rt in the presence of NBS.[2]

2.2 Preparation of diazo compounds 3a–m

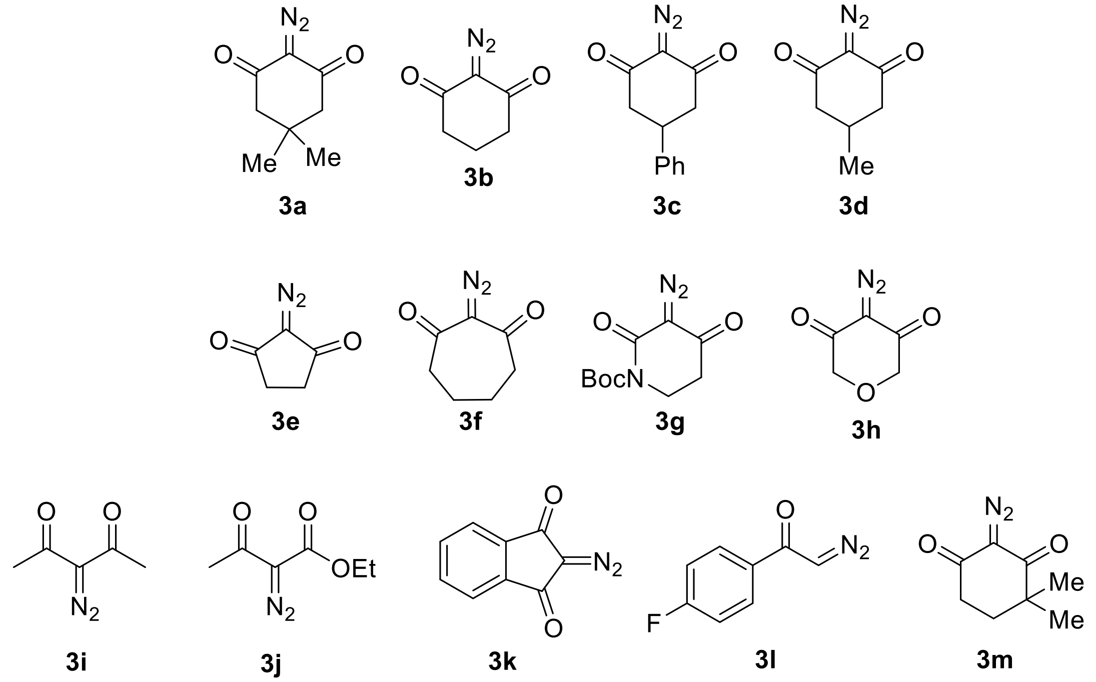

The diazo compounds **3a**–**m** were obtained as described previously.[3]

2.3 Mechanism studies

Preparation of rhodocycle 5

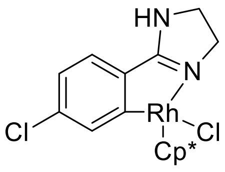

5

2-Arylimidazoline **2a** (0.14 mmol), $[RhCp^*Cl_2]_2$ (0.07 mmol), NaOAc (6.0 equiv), and DCM (2 mL) were charged into a pressure tube. The reaction mixture was stirred at room temperature for 12 h. After filtration, the solvent was removed under reduced pressure and the residue was washed with Et_2O for several times to afford the complex **5**. Yield: 22 mg, 35%. ^{1}H NMR (400 MHz, $CDCl_3$) δ 7.75 – 7.66 (m, 1H), 7.34 – 7.25 (m, 1H), 6.97 – 6.90 (m, 1H), 6.61 (s, 1H), 3.89 – 3.77 (m, 1H), 3.67 – 3.52 (m, 1H), 3.34 – 3.22 (m, 1H), 2.25 – 2.12 (m, 1H), 1.63 (s, 15H). ^{13}C NMR (101 MHz, $CDCl_3$) δ 180.2, 171.8 (d, J = 3.3 Hz), 135.7, 135.7 (d, J = 1.7 Hz), 134.3, 126.0, 122.1, 94.8 (d, J = 6.5 Hz), 51.8, 44.1, 9.5.

^{1}H and ^{13}C NMR of complex

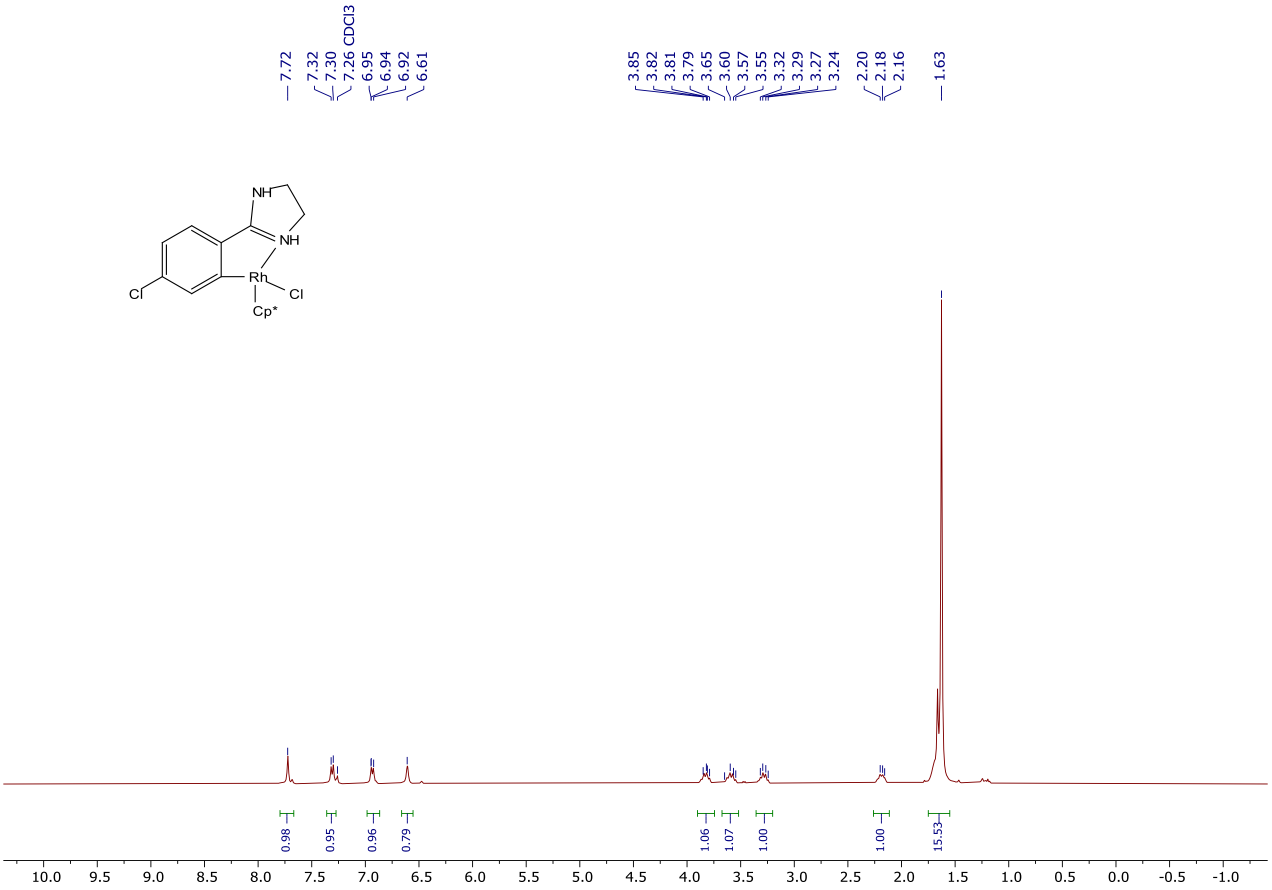

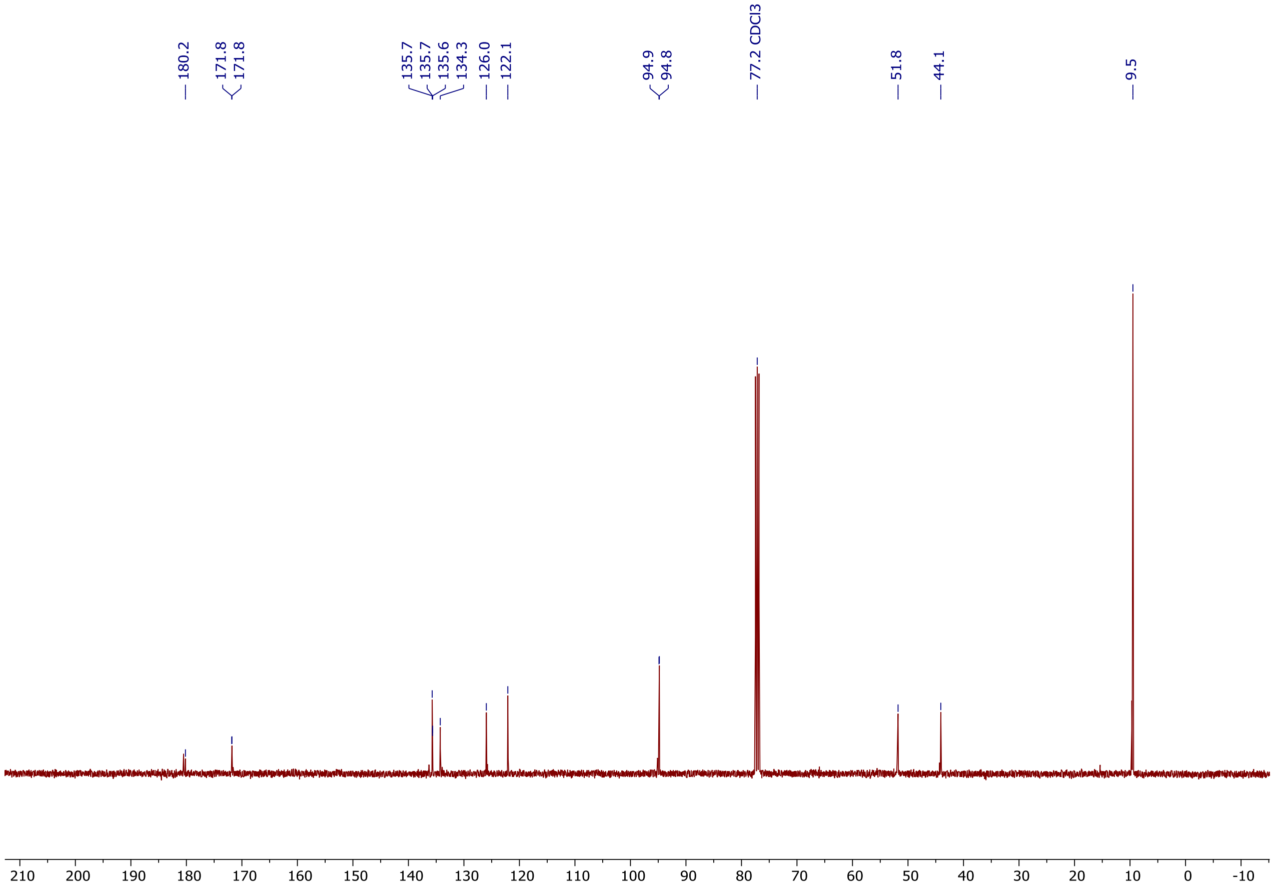

2.4 Preparation of Imidazoisoquinolines 4a–q

<u>**General procedure A (GP1)**</u>**: Preparation of imidazoisoquinoline 4a–q**

A solution of the corresponding imidazoline **2** (0.2 mmol, 1 equiv) and corresponding diazo compound **3** (0.24 mmol, 1.2 equiv) with $[RhCp^*Cl_2]_2$ (2.5 mol %, 0.005 mmol, 1.5 mg) and $AgNTf_2$ (10 mol %, 0.02 mmol, 7.8 mg) in TFE (2 mL) was placed in sealed vial (10 mL) equipped with a stirring bar. The reaction was stirred at 80 °C for 12 h. The solvent was evaporated under reduced pressure and the residue was purified by column chromatography (absorption at λ = 254 nm).

*10-Chloro-6,6-dimethyl-2,5,6,7-tetrahydroimidazo[1,2-f]phenanthridin-8(3H)-one (**4a**).* Obtained according to GP1 from diazo compound **3a** (40 mg, 0.24 mmol, 1.2 equiv) and imidazoline **2a** (36 mg, 0.2 mmol, 1 equiv). Column chromatography was carried out on silica gel, eluent: DCM (with 1% Et_3N)/methanol, from 0 to 5% of methanol. Yield: 53 mg (88%). Pale orange solid; mp 203.8–204.5 °C. ^{1}H NMR (400 MHz, $CDCl_3$) δ 9.30 – 9.24 (m, 1H), 8.02 – 7.96 (m, 1H), 7.28 – 7.22 (m, 1H), 4.17 – 3.98 (m, 4H), 2.53 (s, 2H), 2.38 (s, 2H), 1.12 (s, 6H). ^{13}C NMR (101 MHz, $CDCl_3$) δ 194.5, 155.1, 153.2, 138.8, 134.8, 127.2, 127.0, 126.0, 119.3, 106.5, 53.1, 52.2, 48.0, 42.3, 31.8, 28.5. HRMS (ESI) *m/z*: $[M+H]^+$ Calc. for $C_{17}H_{18}ClN_2O$ 301.1102; Found 301.1105.

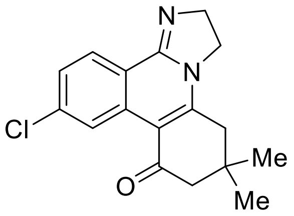

10-Methoxy-6,6-dimethyl-2,5,6,7-tetrahydroimidazo[1,2-f]phenanthridin-8(3H)-one ***(4b)***.

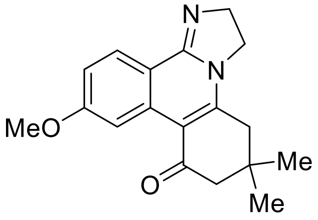

Obtained according to GP1 from diazo compound **3a** (40 mg, 0.24 mmol, 1.2 equiv) and imidazoline **2e** (35 mg, 0.2 mmol, 1 equiv). Column chromatography was carried out on silica gel, eluent: DCM (with 1% Et_3N)/methanol, from 0 to 7% of methanol. Yield: 27 mg (46 %). Yellow solid; mp 235.2–235.7 °C. ^{1}H NMR (400 MHz, $CDCl_3$) δ 8.89 – 8.84 (m, 1H), 8.08 – 8.01 (m, 1H), 6.95 – 6.88 (m, 1H), 4.16 – 3.99 (m, 4H), 3.90 (s, 3H), 2.53 (s, 2H), 2.41 (s, 2H), 1.13 (s, 6H). ^{13}C NMR (101 MHz, $CDCl_3$) δ 195.0, 163.0, 155.4, 152.5, 135.4, 127.4, 115.8, 113.6, 108.1, 107.4, 55.4, 52.3, 52.0, 47.9, 42.1, 31.7, 28.3. HRMS (ESI) *m/z*: $[M+H]^+$ Calc. for $C_{18}H_{21}N_2O_2$ 297.1598; Found 297.1602.

10-Ethyl-6,6-dimethyl-2,5,6,7-tetrahydroimidazo[1,2-f]phenanthridin-8(3H)-one ***(4c)***. Obtained

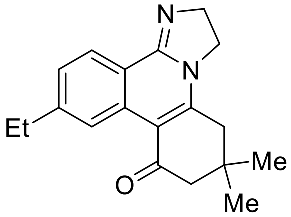

according to GP1 from diazo compound **3a** (40 mg, 0.24 mmol, 1.2 equiv) and imidazoline **2d** (35 mg, 0.2 mmol, 1 equiv). Column chromatography was carried out on silica gel, eluent: DCM (with 1% Et_3N)/methanol, from 0 to 5% of methanol. Yield: 42 mg (71%). Beige solid; mp 215.3–215.9 °C. ^{1}H NMR (400 MHz, $CDCl_3$) δ 9.10 – 9.05 (m, 1H), 8.04 – 7.98 (m, 1H), 7.20 – 7.13 (m, 1H), 4.12 – 3.90 (m, 4H), 2.71 (q, J = 7.6 Hz, 2H), 2.42 (s, 2H), 2.36 (s, 2H), 1.26 (t, J = 7.6 Hz, 3H), 1.08 (s, 6H). ^{13}C NMR (101 MHz, $CDCl_3$) δ 194.9, 155.6, 152.2, 149.0, 133.5, 126.7, 125.8, 125.2, 118.5, 107.2, 52.8, 52.3, 47.8, 42.1, 31.7, 29.5, 28.3, 15.4. HRMS (ESI) *m/z*: $[M+H]^+$ Calc. for $C_{19}H_{23}N_2O$ 295.1805; Found 295.1811.

6,6-Dimethyl-10-nitro-2,5,6,7-tetrahydroimidazo[1,2-f]phenanthridin-8(3H)-one ***(4d)***. Obtained

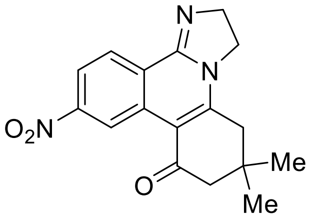

according to GP1 from diazo compound **3a** (40 mg, 0.25 mmol, 1 equiv) and imidazoline **2c** (38 mg, 0.2 mmol, 1 equiv). Column chromatography was carried out on silica gel, eluent: DCM (with 1% Et_3N)/methanol, from 0 to 7% of methanol. Yield: 53 mg (86%). Orange solid; 216.4–216.8 °C. ^{1}H NMR (400 MHz, $CDCl_3$) δ 9.33 – 9.28 (m, 1H), 8.25 – 8.19 (m, 1H), 7.71 – 7.64 (m, 1H), 4.50 (t, J = 9.8 Hz, 2H), 4.12 (t, J = 9.8 Hz, 2H), 2.90 (s, 2H), 2.46 (s, 2H), 1.10 (s, 3H). ^{13}C NMR (101 MHz, $CDCl_3$) δ 195.3, 155.2, 153.5, 139.7, 135.1, 127.9, 127.7, 124.9, 115.2, 108.3, 51.7, 48.3, 47.1, 40.1, 31.6, 27.6. HRMS (ESI) *m/z*: $[M+H]^+$ Calc. for $C_{17}H_{18}N_3O_3$ 312.1343; Found 312.1348.

6,6-Dimethyl-10-(trifluoromethyl)-2,5,6,7-tetrahydroimidazo[1,2-f]phenanthridin-8(3H)-one ***(4e)***.

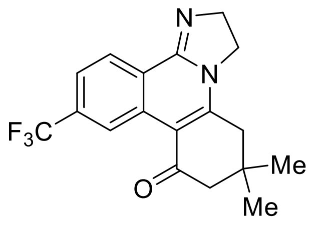

Obtained according to GP1 from diazo compound **3a** (40 mg, 0.24 mmol, 1.2 equiv) and imidazoline **2b** (43 mg, 0.2 mmol, 1 equiv). Column chromatography was carried out on silica gel, eluent: DCM (with 1% Et_3N)/methanol, from 0 to 5% of methanol. Yield: 53 mg (80%). White solid; mp 245.8–246.4 °C. ^{1}H NMR (400 MHz, $CDCl_3$) δ 9.63 – 9.59 (m, 1H), 8.26 – 8.19 (m, 1H), 7.58 – 7.51 (m, 1H), 4.26 – 4.09 (m, 4H), 2.64 (s, 2H), 2.46 (s, 2H), 1.17 (s, 6H). ^{13}C NMR (101 MHz, $CDCl_3$) δ 194.7, 155.1, 153.2, 133.9 (q, *J* = 32.1 Hz), 133.9, 126.5, 124.1 (q, *J* = 273.0 Hz), 123.8 (q, *J* = 4.2 Hz), 123.3 – 123.3 (m), 122.9 (q, *J* = 3.5 Hz), 106.9, 53.1, 52.3, 48.1, 42.4, 31.9, 28.5. ^{19}F NMR (376 MHz, $CDCl_3$) δ -63.02. HRMS (ESI) *m/z*: $[M+H]^+$ Calc. for $C_{17}H_{18}ClN_2O$ 301.1102; Found 301.1107.

12-Fluoro-6,6-dimethyl-2,5,6,7-tetrahydroimidazo[1,2-f]phenanthridin-8(3H)-one ***(4f)***. Obtained

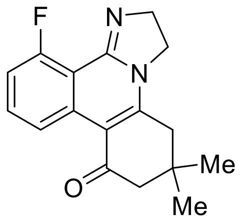

according to GP1 from diazo compound **3a** (40 mg, 0.24 mmol, 1.2 equiv) and imidazoline **2f** (33 mg, 0.2 mmol, 1 equiv). Column chromatography was carried out on silica gel, eluent: DCM (with 1% Et_3N)/methanol, from 0 to 5% of methanol. Yield: 57 mg (99%). White solid; 215.3–217.3 °C. ^{1}H NMR (400 MHz, $CDCl_3$) δ 9.07 – 9.01 (m, 1H), 7.51 – 7.41 (m, 1H), 7.07 – 6.97 (m, 1H), 4.26 – 4.15 (m, 2H), 4.01 – 3.92 (m, 2H), 2.55 (s, 2H), 2.40 (s, 2H), 1.12 (s, 3H). ^{13}C NMR (101 MHz, $CDCl_3$) δ 194.4, 160.9 (d, *J* = 259.4 Hz), 153.1, 152.2 (d, *J* = 8.4 Hz), 135. 9 (d, *J* = 1.0 Hz), 133.0 (d, *J* = 9.6 Hz), 121.8 (d, *J* = 4.3 Hz), 113.6 (d, *J* = 21.0 Hz), 110.3 (d, *J* = 9.0 Hz), 106.8 (d, *J* = 1.8 Hz), 53.8, 52.3, 46.7, 42.5, 31.6, 28.4. ^{19}F NMR (376 MHz, $CDCl_3$) δ -108.38. HRMS (ESI) *m/z*: $[M+H]^+$ Calc. for $C_{17}H_{18}FN_2O$ 285.1398; Found 285.1403.

2,6,6-Trimethyl-2,5,6,7-tetrahydroimidazo[1,2-f]phenanthridin-8(3H)-one ***(4g)***. Obtained

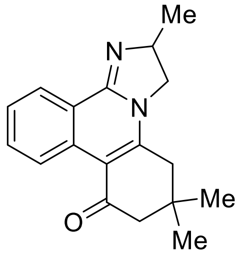

according to GP1 from diazo compound **3a** (40 mg, 0.24 mmol, 1.2 equiv) and imidazoline **2g** (32 mg, 0.2 mmol, 1 equiv). Column chromatography was carried out on silica gel, eluent: DCM (with 1% Et_3N)/methanol, from 0 to 10% of methanol. Yield: 43 mg (77%). Beige solid. Mixture of regioisomers in ratio 87:13. *Major regioisomer*: ^{1}H NMR (400 MHz, $CDCl_3$) δ 9.23 – 9.16 (m, 1H), 8.15 – 8.08 (m, 1H), 7.58 – 7.49 (m, 1H), 7.36 – 7.24 (m, 1H), 4.52 – 4.35 (m, 1H), 4.15 (t, *J* = 10.3 Hz, 1H), 3.58 (dd, *J* = 10.5, 7.5 Hz, 1H), 2.52 (s, 2H), 2.39 (s, 2H), 1.40 (d, *J* = 6.6 Hz, 3H), 1.11 (s, 6H). ^{13}C NMR (101 MHz, $CDCl_3$) δ 194.9, 154.5, 152.1, 133.5, 132.3, 126.6, 126.3, 125.9, 120.7, 107.5, 59.9, 54.8, 52.4, 42.2, 31.8, 28.5, 22.9. *Minor regioisomer*: ^{1}H NMR (400 MHz, $CDCl_3$) δ 9.23 – 9.16 (m, 1H), 8.15 – 8.08 (m, 1H), 7.58 – 7.49 (m, 1H), 7.36 – 7.24 (m, 1H), 4.52 – 4.35 (m, 1H), 4.23 – 4.18 (m, 1H), 3.72 (dd, *J* = 14.8, 2.8 Hz, 1H), 2.43 (s, 2H), 2.41 (s, 2H), 1.36 (d, *J* = 6.3

Hz, 3H), 1.14 (s, 6H). ^{13}C NMR (101 MHz, $CDCl_3$) δ 194.9, 154.8, 151.9, 133.3, 132.3, 126.7, 126.4, 125.7, 121.4, 108.0, 60.9, 55.7, 52.7, 41.3, 32.2, 28.1, 21.8. HRMS (ESI) *m/z*: $[M+H]^+$ Calc. for $C_{18}H_{21}N_2O$ 281.1648; Found 281.1651.

10-Chloro-2,5,6,7-tetrahydroimidazo[1,2-f]phenanthridin-8(3H)-one ***(4h)***. Obtained according to GP1 from diazo compound **3b** (33 mg, 0.24 mmol, 1.2 equiv) and imidazoline **2a** (36 mg, 0.2 mmol, 1 equiv). Column chromatography was carried out on silica gel, eluent: DCM (with 1% Et_3N)/methanol, from 0 to 5% of methanol. Yield: 48 mg (89%). Pale orange solid; mp 176.0–176.2 °C. ^{1}H NMR (400 MHz, $CDCl_3$) δ 9.23 – 9.18 (m, 1H), 7.96 – 7.90 (m, 2H), 7.30 – 7.19 (m, 2H), 4.13 – 3.94 (m, 6H), 2.65 (t, J = 6.3 Hz, 4H), 2.49 (t, J = 6.6 Hz, 3H), 2.05 (p, J = 6.4 Hz, 3H). ^{13}C NMR (101 MHz, $CDCl_3$) δ 194.6, 154.7, 154.7, 138.5, 134.9, 127.2, 126.8, 126.1, 119.3, 107.4, 53.0, 47.8, 38.5, 28.6, 20.5. HRMS (ESI) *m/z*: $[M+H]^+$ Calc. for $C_{15}H_{14}ClN_2O$ 273.0789; Found 273.0793.

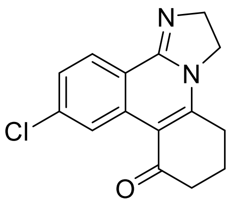

10-(Trifluoromethyl)-2,5,6,7-tetrahydroimidazo[1,2-f]phenanthridin-8(3H)-one ***(4i)***. Obtained according to GP1 from diazo compound **3b** (33 mg, 0.24 mmol, 1.2 equiv) and imidazoline **2b** (43 mg, 0.2 mmol, 1 equiv). Column chromatography was carried out on silica gel, eluent: DCM (with 1% Et_3N)/methanol, from 0 to 10% of methanol. Yield: 45 mg (74%). White solid; mp 232.4–233.0 °C. ^{1}H NMR (400 MHz, $CDCl_3$) δ 9.61 – 9.56 (m, 1H), 8.24 – 8.18 (m, 1H), 7.59 – 7.52 (m, 1H), 4.25 – 4.07 (m, 4H), 2.78 (t, J = 6.3 Hz, 2H), 2.58 (t, J = 6.4 Hz, 2H), 2.13 (p, J = 6.4 Hz, 2H). ^{13}C NMR (101 MHz, $CDCl_3$) δ 194.7, 154.9, 154.7, 134.0, 133.8 (q, J = 32.1 Hz), 126.5, 124.1 (q, J = 273.1 Hz), 124.1 (q, J = 4.1 Hz), 123.6 – 123.5 (m), 122.9 (q, J = 3.6 Hz), 107.9, 53.2, 48.0, 38.6, 28.7, 20.6. ^{19}F NMR (376 MHz, $CDCl_3$) δ -63.00. HRMS (ESI) *m/z*: $[M+H]^+$ Calc. for $C_{16}H_{14}F_3N_2O$ 307.1053; Found 307.1058.

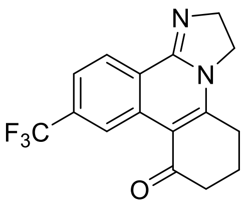

10-Nitro-2,5,6,7-tetrahydroimidazo[1,2-f]phenanthridin-8(3H)-one ***(4j)***. Obtained according to GP1 from diazo compound **3b** (33 mg, 0.24 mmol, 1.2 equiv) and imidazoline **2c** (38 mg, 0.2 mmol, 1 equiv). Column chromatography was carried out on silica gel, eluent: DCM (with 1% Et_3N)/methanol, from 0 to 7% of methanol. Yield: 43 mg (77%). Orange solid. ^{1}H NMR (400 MHz, $CDCl_3$) δ 10.09 – 10.04 (m, 1H), 8.21 – 8.14 (m, 1H), 8.09 – 8.02 (m, 1H), 4.25 – 4.08 (m, 4H), 2.80 (t, J = 6.1 Hz, 2H), 2.59 (t, J = 6.5 Hz, 2H), 2.15 (p, J = 6.8 Hz, 2H). ^{13}C NMR (101 MHz, $CDCl_3$) δ 194.4, 155.4, 154.3, 150.2, 134.6, 127.0, 125.4, 123.8, 122.1, 120.6, 107.5, 53.5, 48.0, 38.5, 28.7, 20.5. HRMS (ESI) *m/z*: $[M+H]^+$ Calc. for $C_{15}H_{14}N_3O_3$ 284.1030; Found 284.1034.

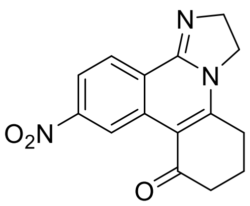

10-Methoxy-6-phenyl-2,5,6,7-tetrahydroimidazo[1,2-f]phenanthridin-8(3H)-one ***(4k)***. Obtained according to GP1 from diazo compound **3c** (52 mg, 0.24 mmol, 1.2 equiv) and imidazoline **2e** (38 mg, 0.2 mmol, 1 equiv). Column chromatography was carried out on silica gel, eluent: DCM (with 1% Et_3N)/methanol, from 0 to 3% of methanol. Yield: 64 mg (93%). Beige solid. ^{1}H NMR (400 MHz, $CDCl_3$) δ 8.91 – 8.85 (m, 1H), 8.09 – 8.03 (m, 1H), 7.44 – 7.36 (m, 2H), 7.34 – 7.28 (m, 3H), 6.98 – 6.91 (m, 1H), 4.18 – 4.06 (m, 3H), 4.04 – 3.98 (m, 1H), 3.92 (s, 3H), 3.54 – 3.41 (m, 1H), 3.01 – 2.91 (m, 2H), 2.86 – 2.79 (m, 2H). ^{13}C NMR (101 MHz, $CDCl_3$) δ 194.2, 162.8, 154.9, 153.8, 142.4, 135.4, 129.0, 127.5, 127.4, 115.5, 114.1, 108.5, 107.5, 55.4, 52.7, 47.8, 45.4, 38.5, 36.1. HRMS (ESI) *m/z*: $[M+H]^+$ Calc. for $C_{22}H_{21}N_2O_2$ 345.1598; Found 345.1602.

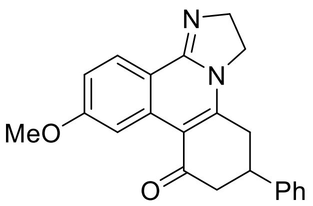

10-Chloro-6-phenyl-2,5,6,7-tetrahydroimidazo[1,2-f]phenanthridin-8(3H)-one ***(4l)***. Obtained according to GP1 from diazo compound **3c** (52 mg, 0.24 mmol, 1.2 equiv) and imidazoline **2a** (36 mg, 0.2 mmol, 1 equiv). Column chromatography was carried out on silica gel, eluent: DCM (with 1% Et_3N)/methanol, from 0 to 3% of methanol. Yield: 63 mg (91%). White solid; mp 234.4–234.7 °C. ^{1}H NMR (400 MHz, $CDCl_3$) δ 9.33 – 9.28 (m, 1H), 8.07 – 8.00 (m, 1H), 7.44 – 7.36 (m, 1H), 7.31 – 7.24 (m, 1H), 4.30 – 3.93 (m, 2H), 3.52 – 3.39 (m, 1H), 3.01 – 2.84 (m, 1H), 2.83 – 2.73 (m, 2H). ^{13}C NMR (101 MHz, $CDCl_3$) δ 193.8, 154.8, 153.9, 142.2, 138.9, 134.8, 129.2, 127.6, 127.3, 127.2, 126.2, 119.4, 107.2, 53.1, 48.0, 45.4, 38.6, 36.4. HRMS (ESI) *m/z*: $[M+H]^+$ Calc. for $C_{21}H_{18}ClN_2O$ 349.1102; Found 349.1107.

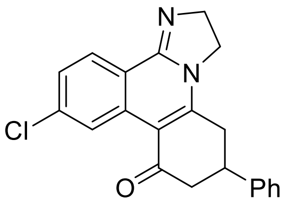

10-Chloro-2,5-dihydro-3H-imidazo[2,1-a]pyrano[3,4-c]isoquinolin-8(7H)-one ***(4m)***. Obtained according to GP1 from diazo compound **3h** (34 mg, 0.24 mmol, 1.2 equiv) and imidazoline **2a** (36 mg, 0.2 mmol, 1 equiv). Column chromatography was carried out on silica gel, eluent: DCM (with 1% Et_3N)/methanol, from 0 to 13% of methanol. Yield: 32 mg (64%). Yellow solid; mp 180.4–181.7 °C. ^{1}H NMR (400 MHz, $CDCl_3$) δ 9.05 – 9.00 (m, 1H), 7.98 – 7.92 (m, 1H), 7.32 – 7.25 (m, 1H), 4.58 (s, 1H), 4.21 – 4.12 (m, 2H), 3.93 (t, J = 9.6 Hz, 1H). ^{13}C NMR (101 MHz, $CDCl_3$) δ 189.3, 154.1, 151.8, 138.9, 133.3, 127.5, 127.3, 125.8, 119.3, 72.2, 64.4, 53.7, 46.4. HRMS (ESI) *m/z*: $[M+H]^+$ Calc. for $C_{14}H_{12}ClN_2O_2$ 275.0582; Found 275.0585.

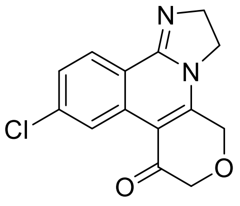

11-Chloro-2,3,5,6,7,8-hexahydro-9H-cyclohepta[c]imidazo[2,1-a]isoquinolin-9-one ***(4n)***. Obtained according to GP1 from diazo compound **3f** (37 mg, 0.24 mmol, 1.2 equiv) and imidazoline **2a** (36 mg, 0.2 mmol, 1 equiv). Column chromatography was carried out on silica gel, eluent: DCM (with 1% Et_3N)/methanol, from 0 to 10% of methanol. Yield: 34 mg (60%). Pale yellow

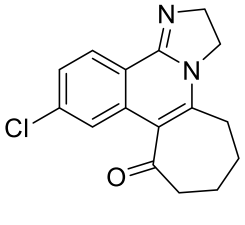

solid; mp 168.3–168.8 °C. ^{1}H NMR (400 MHz, $CDCl_3$) δ 8.20 – 8.15 (m, 1H), 7.99 – 7.92 (m, 1H), 7.25 – 7.16 (m, 1H), 4.14 – 3.99 (m, 3H), 2.73 (t, *J* = 6.5 Hz, 1H), 2.59 (t, *J* = 6.1 Hz, 1H), 1.88 – 1.76 (m, 3H). ^{13}C NMR (101 MHz, $CDCl_3$) δ 203.4, 155.6, 148.2, 138.9, 135.1, 127.6, 126.9, 124.2, 118.5, 112.6, 51.4, 48.3, 42.0, 29.2, 22.2, 20.6. HRMS (ESI) *m/z*: $[M+H]^+$ Calc. for $C_{16}H_{16}ClN_2O$ 273.0789; Found 273.0792.

*9-Chloro-2,3,5,6-tetrahydro-7H-cyclopenta[c]imidazo[2,1-a]isoquinolin-7-one (**4o**).* Obtained according to GP1 from diazo compound **3e** (30 mg, 0.24 mmol, 1.2 equiv) and imidazoline **2a** (36 mg, 0.2 mmol, 1 equiv). Column chromatography was carried out on silica gel, eluent: DCM (with 1% Et_3N)/methanol, from 0 to 15% of methanol. Yield: 45 mg (87%). White solid; mp 243.9–244.6 °C. ^{1}H NMR (400 MHz, DMSO-d_6+$CDCl_3$ in ratio 1:6) δ 8.46 – 8.41 (m, 1H), 7.51 – 7.47 (m, 1H), 7.24 – 7.17 (m, 1H), 4.19 – 4.09 (m, 2H), 4.06 – 3.96 (m, 2H), 2.81 – 2.74 (m, 2H), 2.58 – 2.51 (m, 2H). ^{13}C NMR (101 MHz, DMSO-d_6+$CDCl_3$ in ratio 1:6) δ 197.8, 165.5, 154.8, 137.8, 132.1, 127.1, 126.5, 122.0, 118.5, 109.8, 53.6, 45.1, 34.1, 23.7. HRMS (ESI) *m/z*: $[M+H]^+$ Calc. for $C_{14}H_{12}ClN_2O$ 259.0633; Found 259.0638.

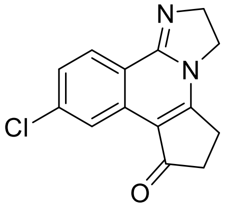

*tert-Butyl 10-hloro-8-oxo-2,3,5,8-tetrahydrobenzo[c]imidazo[1,2-a][1,6]naphthyridine-7(6H)-carboxylate (**4p**).* Obtained according to GP1 from diazo compound **3g** (57 mg, 0.24 mmol, 1.2 equiv) and imidazoline **2a** (36 mg, 0.2 mmol, 1 equiv). Column chromatography was carried out on silica gel, eluent: DCM (with 1% Et_3N)/methanol, from 0 to 15% of methanol. Yield: 54 mg (72%). Beige solid; mp 137.6–138.9 °C. ^{1}H NMR (400 MHz, $CDCl_3$) δ 8.96 – 8.91 (m, 1H), 7.94 – 7.88 (m, 1H), 7.28 – 7.20 (m, 1H), 4.14 – 3.97 (m, 4H), 3.91 (t, *J* = 6.3 Hz, 2H), 2.77 (t, *J* = 6.3 Hz, 2H), 1.56 (s, 9H). ^{13}C NMR (101 MHz, $CDCl_3$) δ 162.94, 154.7, 152.7, 149.6, 138.7, 134.9, 127.3, 127.0, 126.2, 119.7, 101.6, 83.1, 53.1, 47.5, 41.2, 28.3, 27.4. HRMS (ESI) *m/z*: $[M+H]^+$ Calc. for $C_{14}H_{13}ClN_3O$ 374.1266; Found 374.1271.

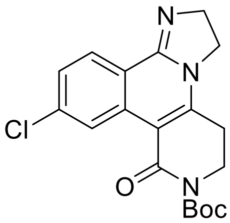

*10-Chloro-6-methyl-2,5,6,7-tetrahydroimidazo[1,2-f]phenanthridin-8(3H)-one (**4q**).* Obtained according to GP1 from diazo compound **3d** (37 mg, 0.24 mmol, 1.2 equiv) and imidazoline **2a** (36 mg, 0.2 mmol, 1 equiv). Column chromatography was carried out on silica gel, eluent: DCM (with 1% Et_3N)/methanol, from 0 to 7% of methanol. Yield: 35 mg (61%). White solid; mp 168.0–168.4 °C. ^{1}H NMR (400 MHz, $CDCl_3$) δ 9.26 – 9.21 (m, 1H), 8.02 – 7.92 (m, 1H), 7.28 – 7.20 (m, 1H),

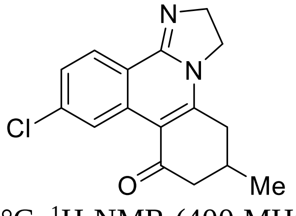

4.16 – 3.91 (m, 3H), 2.73 – 2.60 (m, 1H), 2.59 – 2.51 (m, 1H), 2.38 – 2.14 (m, 1H), 1.12 (d, *J* = 6.1 Hz, 2H). ^{13}C NMR (101 MHz, $CDCl_3$) δ 194.6, 154.9, 154.2, 138.75, 134.9, 127.2, 126.9, 126.1, 119.3, 107.1, 53.0, 47.9, 46.75, 36.6, 27.9, 21.1. HRMS (ESI) *m/z*: $[M+H]^+$ Calc. for $C_{16}H_{16}ClN_2O$ 273.0789; Found 273.0793.

2. Copies of NMR spectra

Copy of ^{1}H (400.13 MHz, $CDCl_3$) NMR spectrum of **4a**

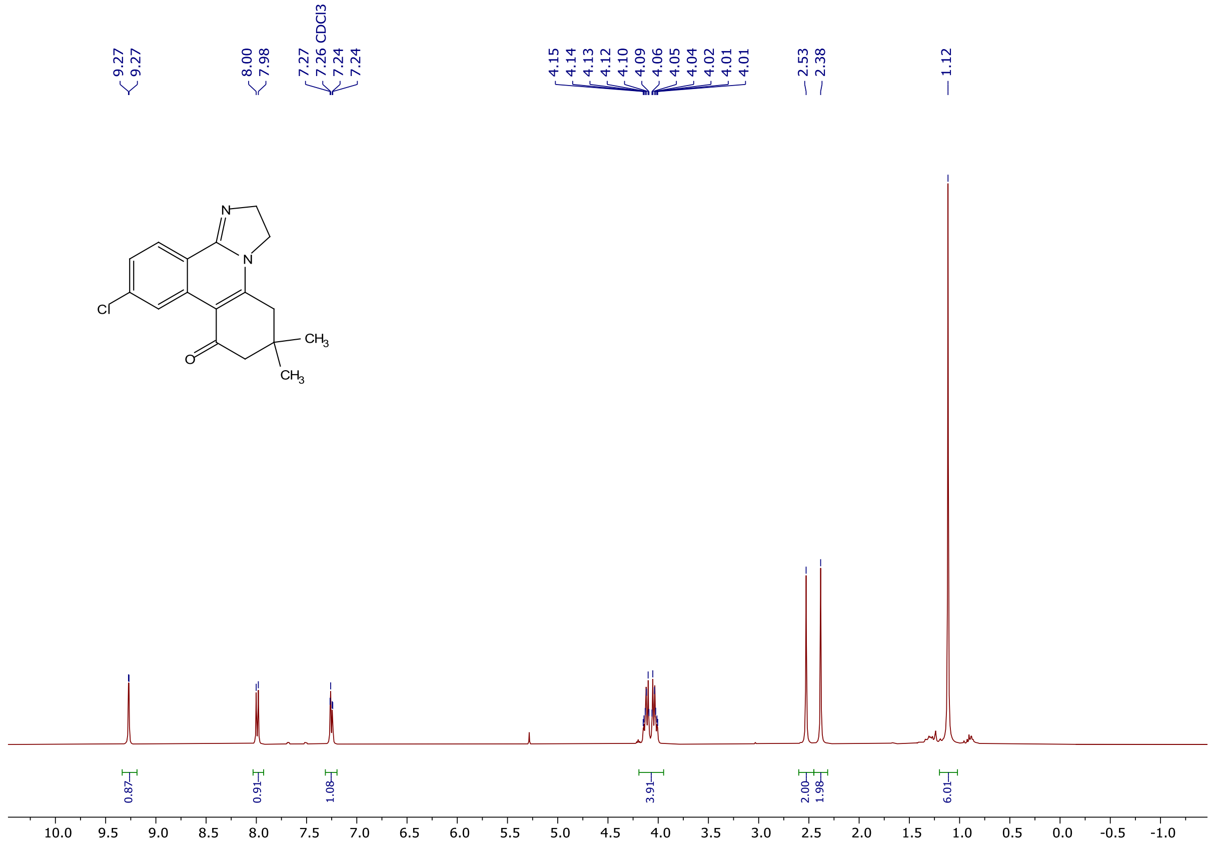

Copy of $^{13}C\{^{1}H\}$ (100.61 MHz, $CDCl_3$) NMR spectrum of **4a**

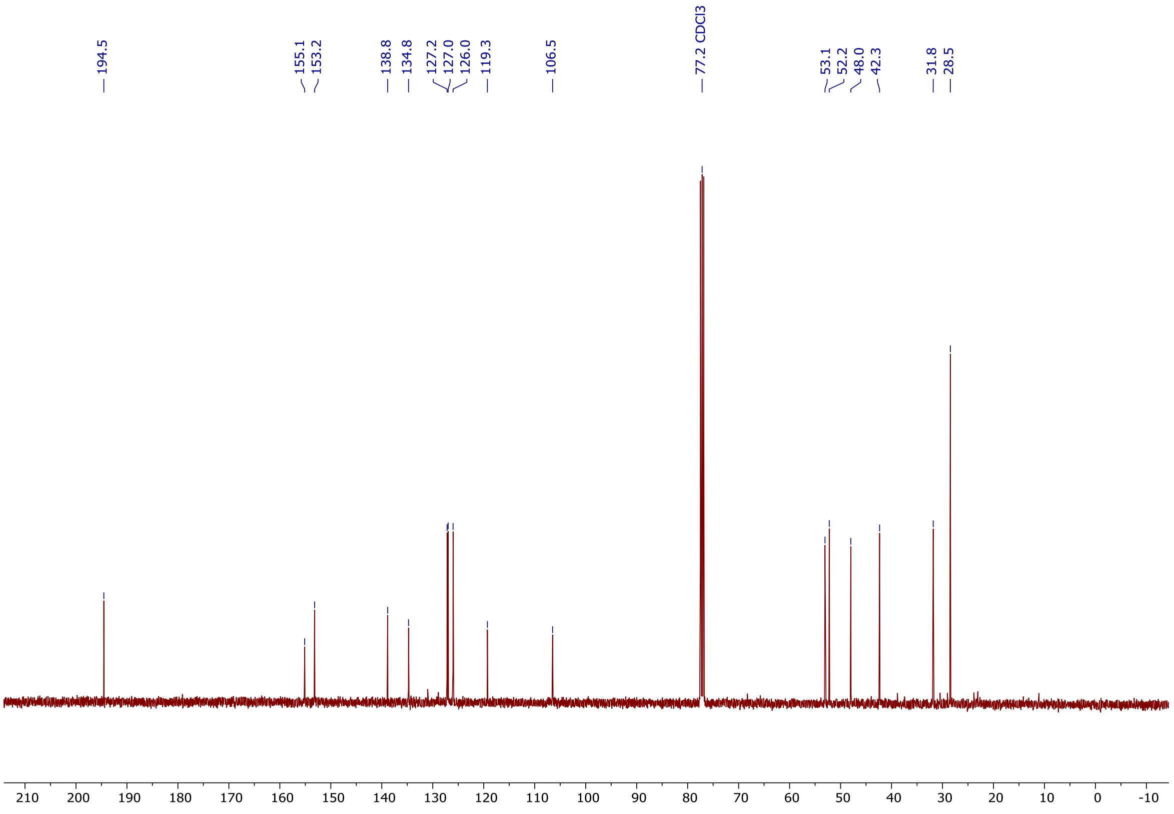

Copy of ^{1}H (400.13 MHz, $CDCl_3$) NMR spectrum of **4b**

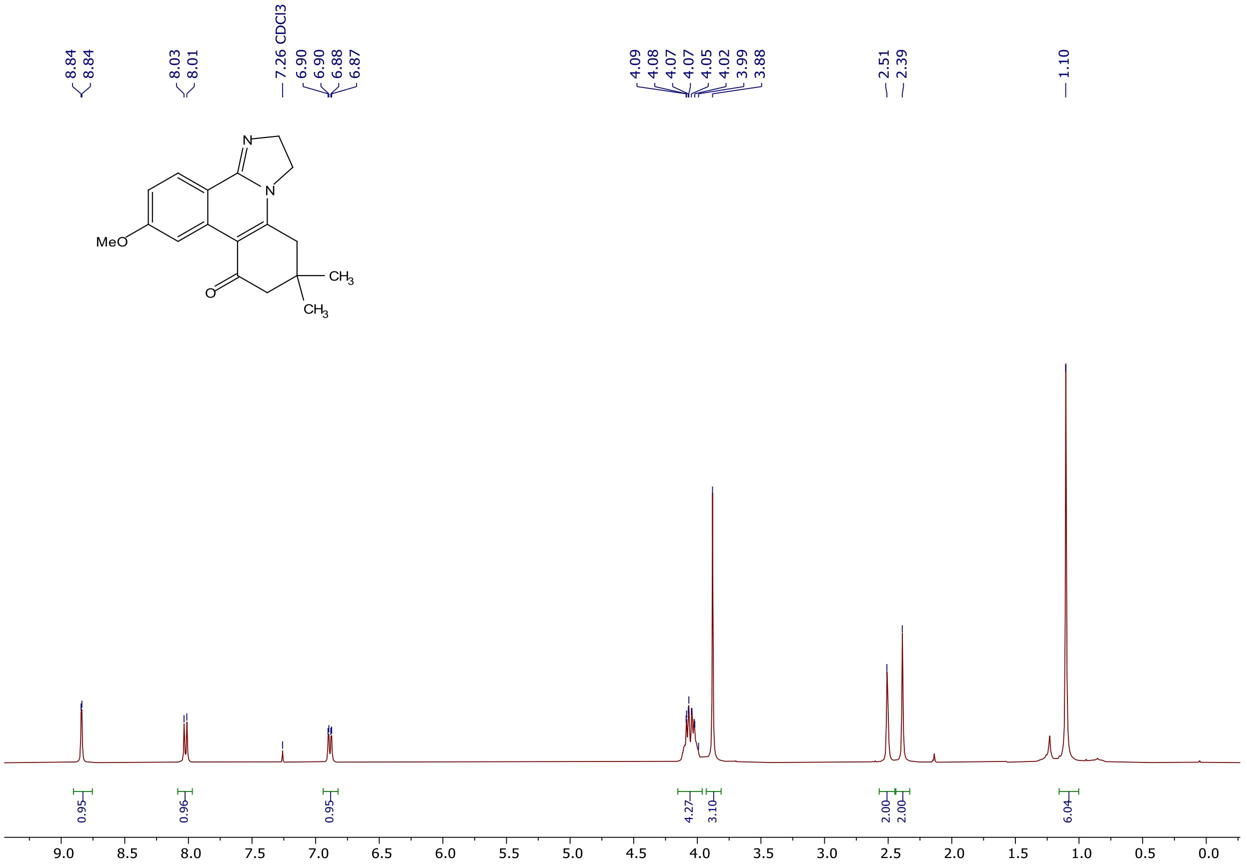

Copy of $^{13}C\{^{1}H\}$ (100.61 MHz, $CDCl_3$) NMR spectrum of **4b**

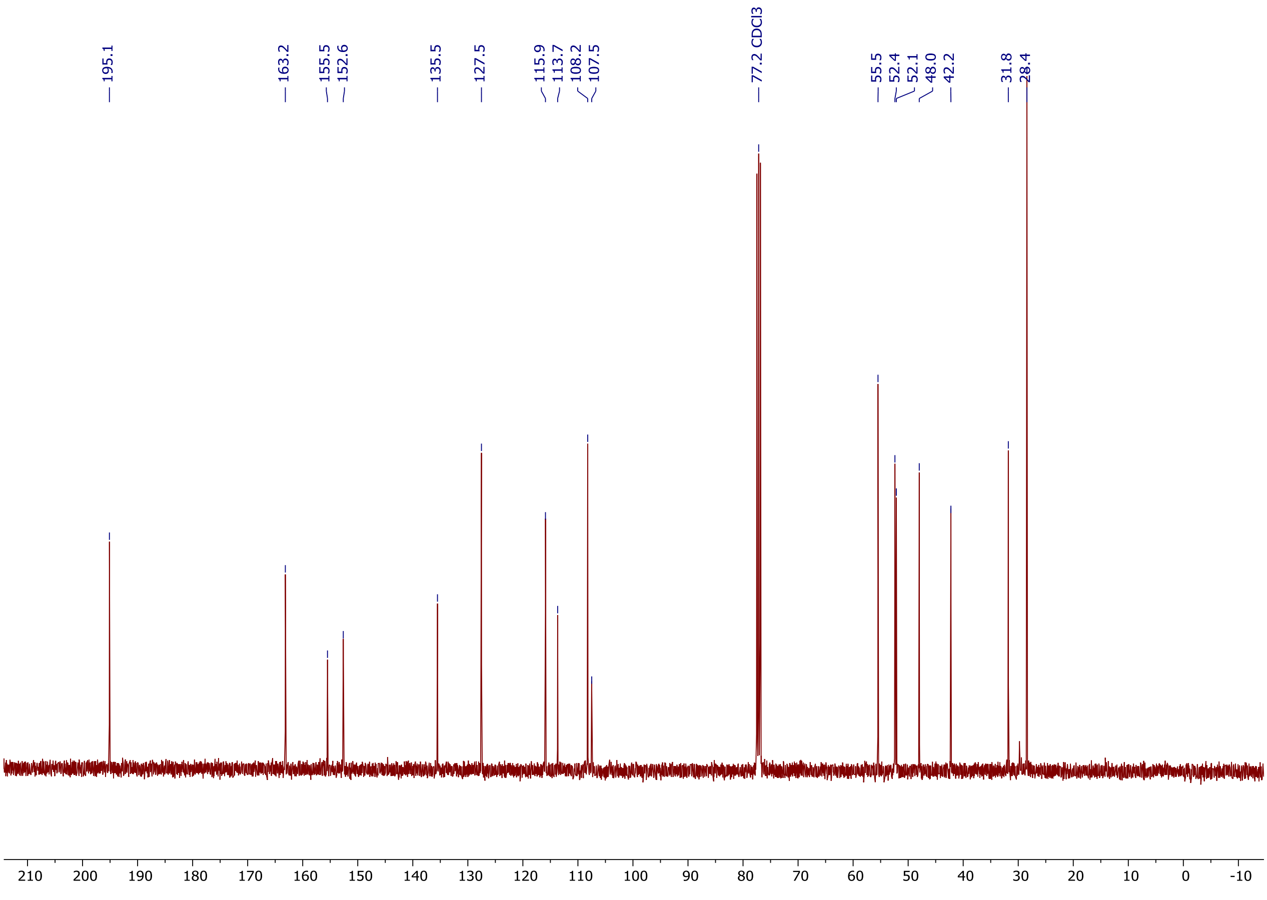

Copy of ^{1}H (400.13 MHz, $CDCl_3$) NMR spectrum of **4c**

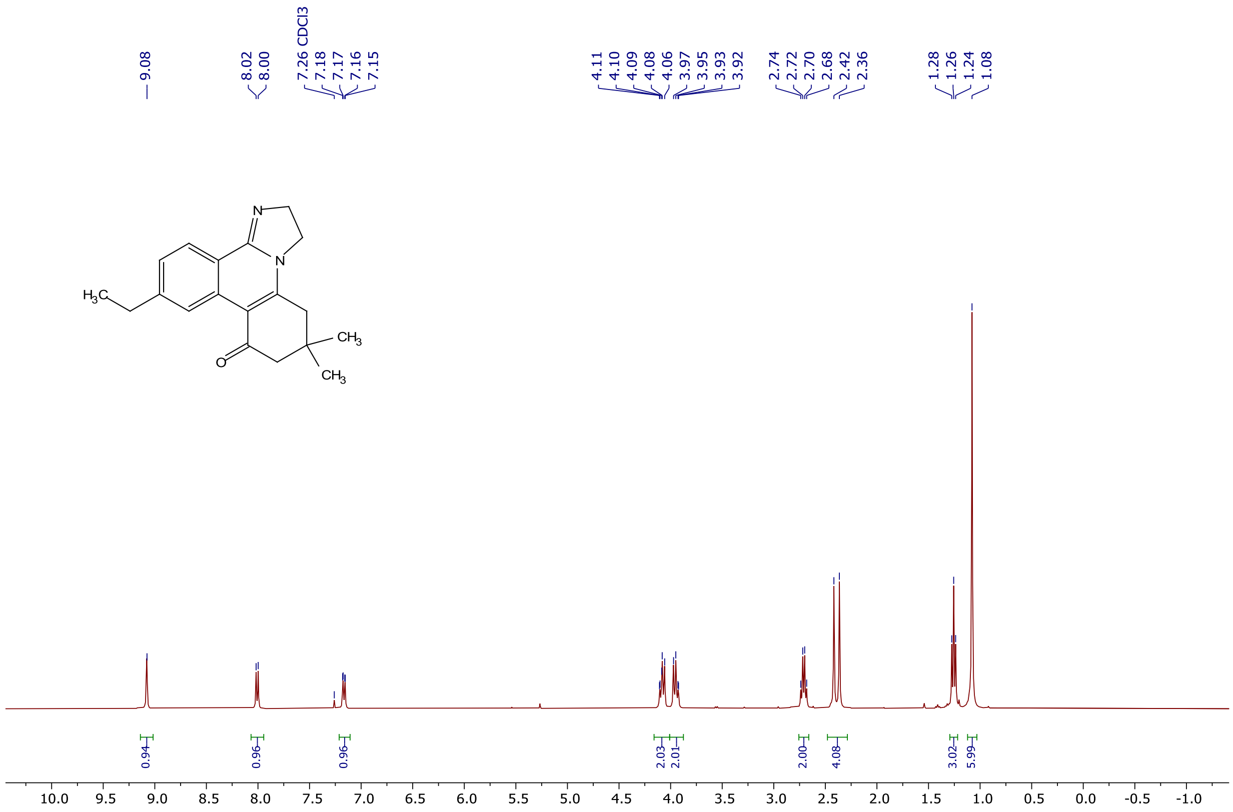

Copy of $^{13}C\{^{1}H\}$ (100.61 MHz, $CDCl_3$) NMR spectrum of **4c**

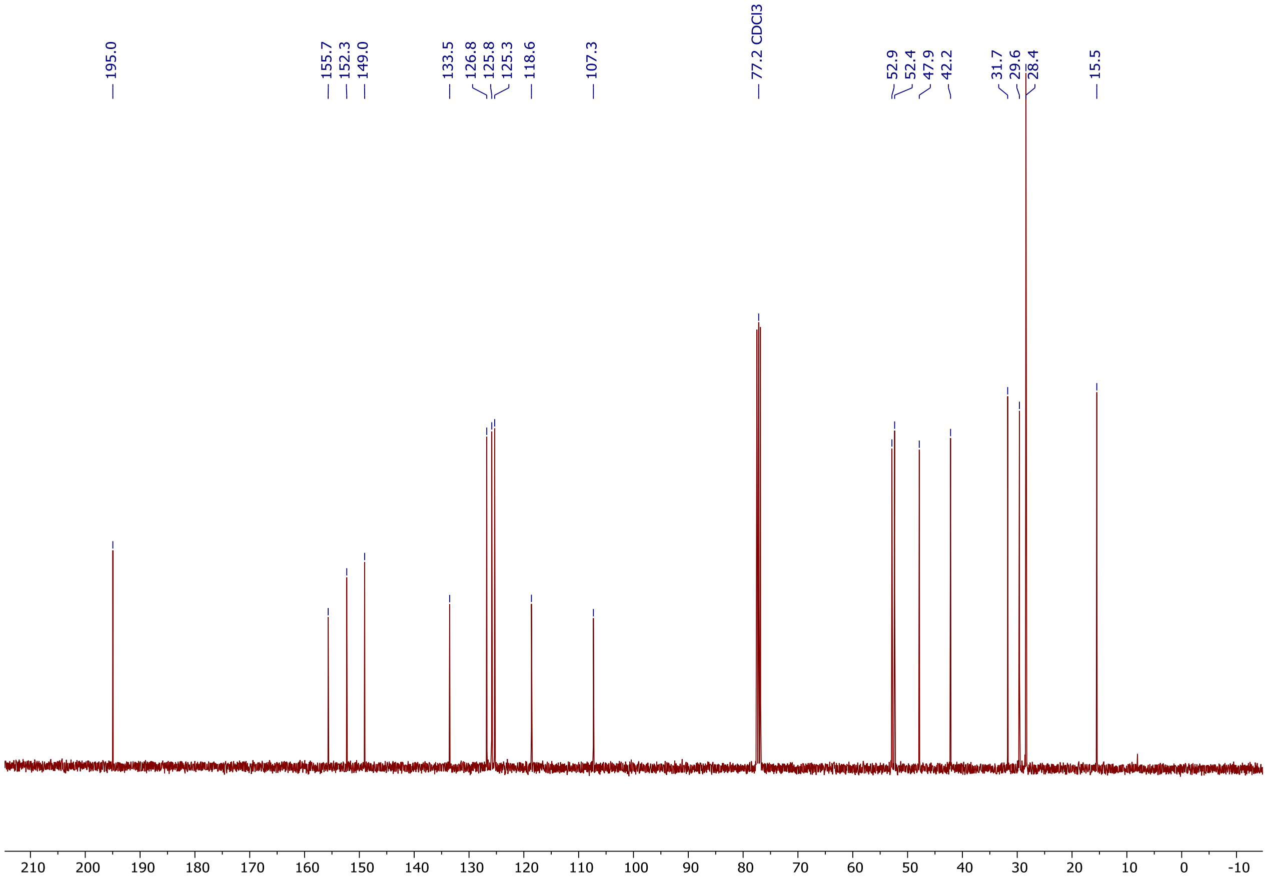

Copy of ^{1}H (400.13 MHz, DMSO-d_6) NMR spectrum of **4d**

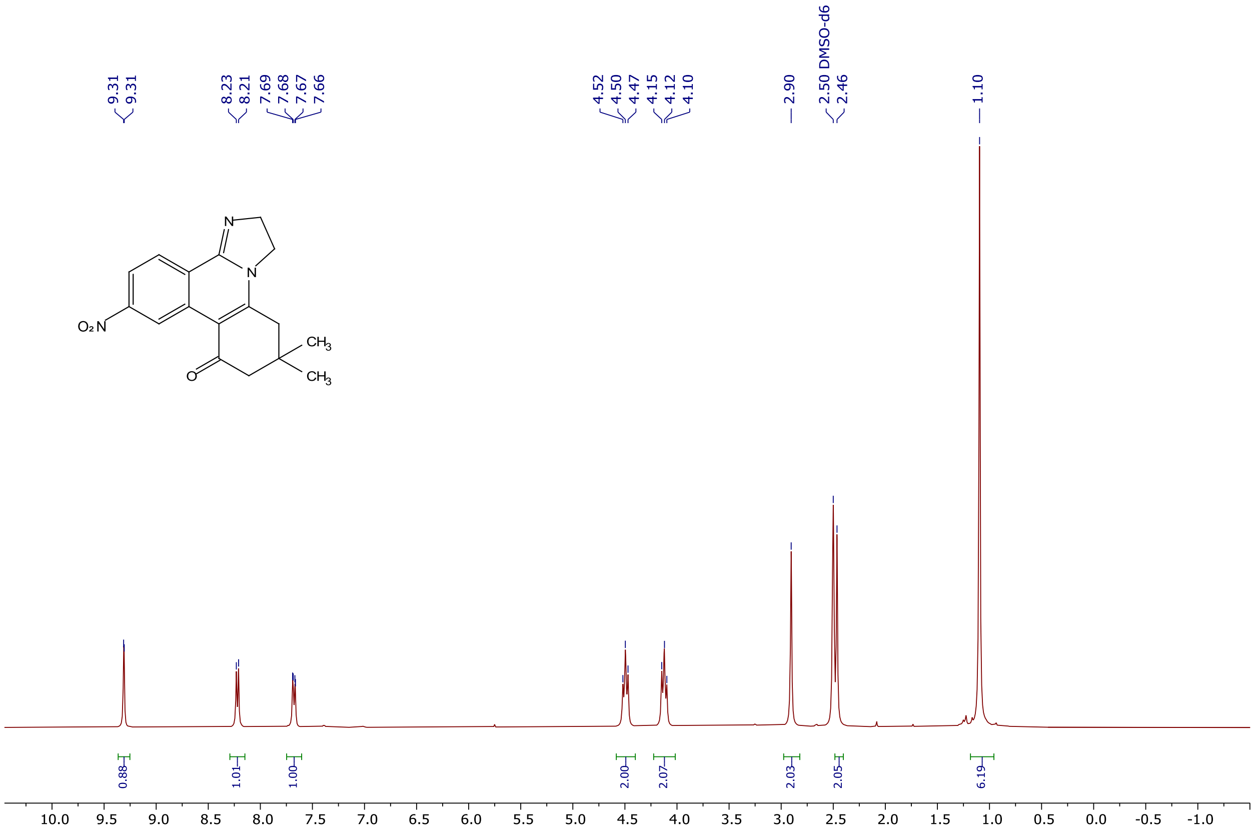

Copy of $^{13}C\{^{1}H\}$ (100.61 MHz, DMSO-d_6) NMR spectrum of **4d**

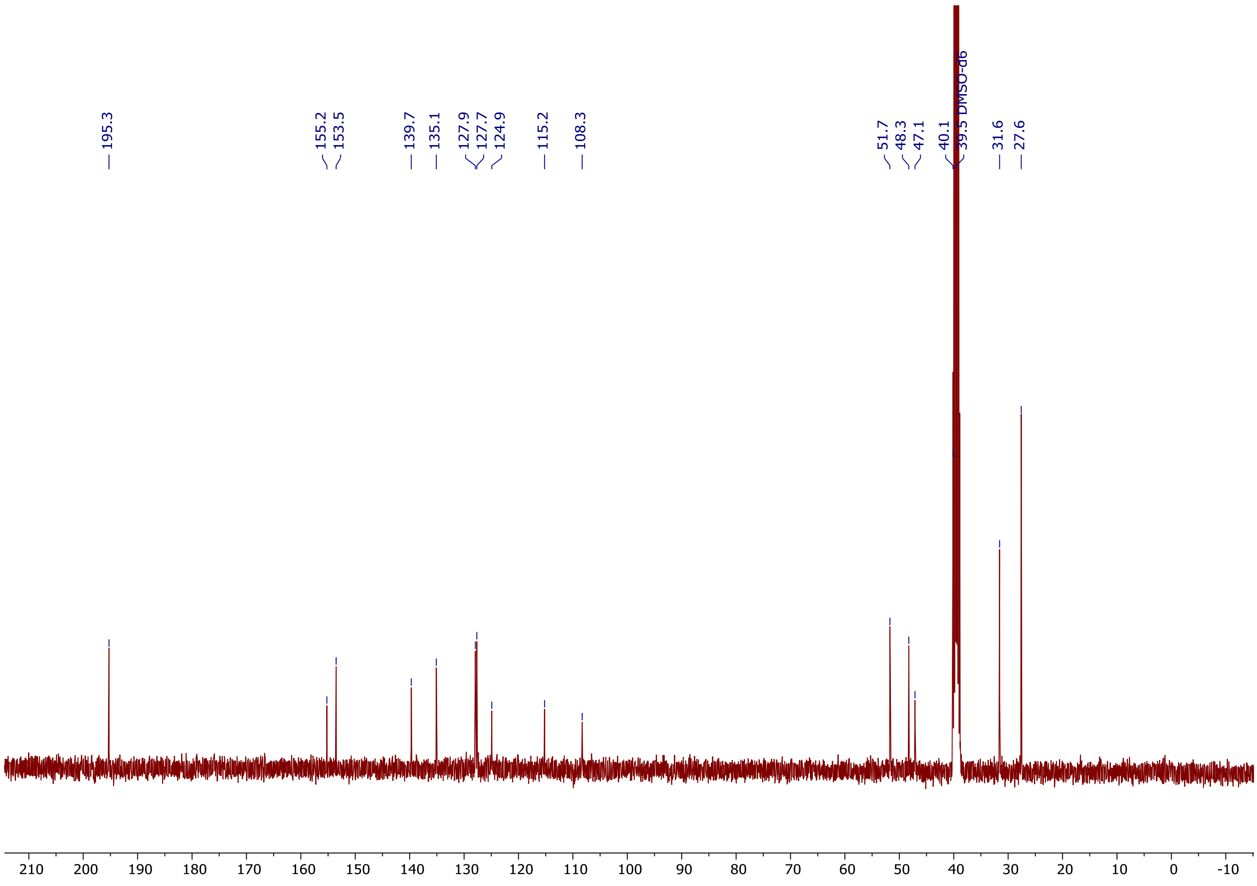

Copy of ^{1}H (400.13 MHz, $CDCl_3$) NMR spectrum of **4e**

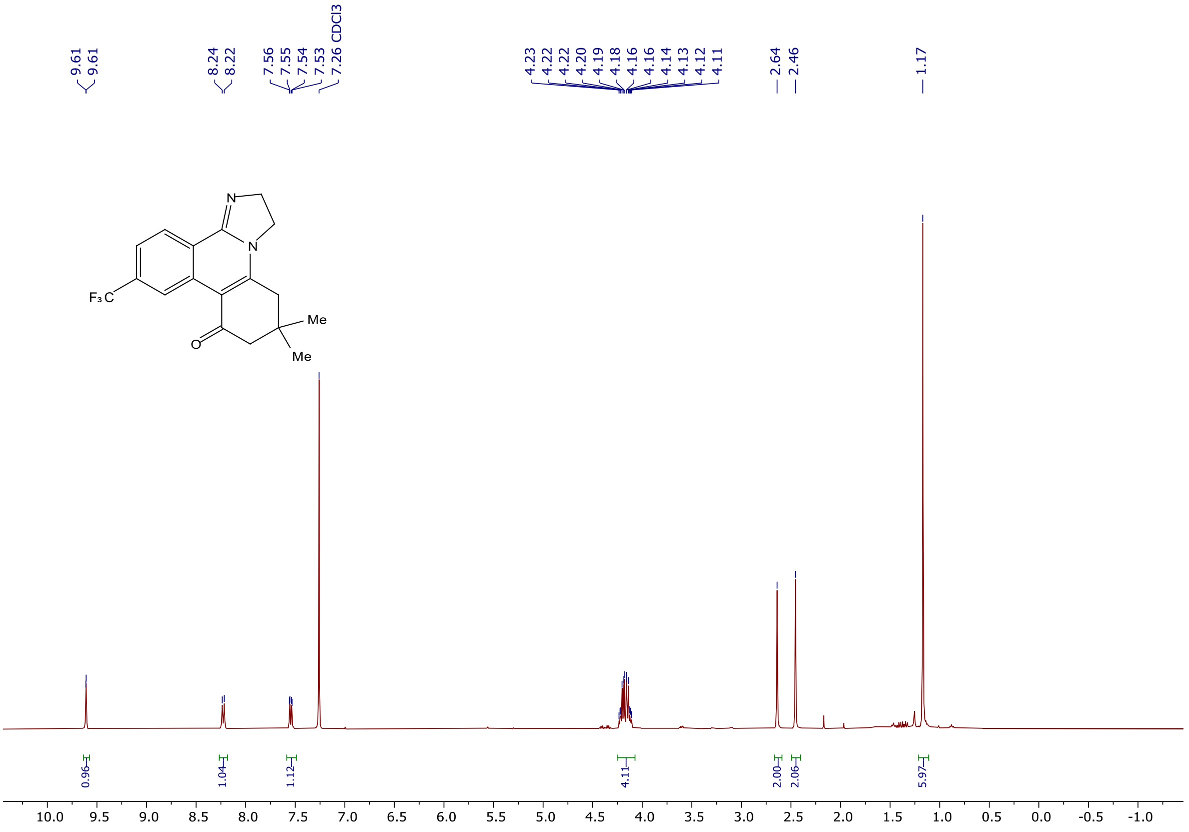

Copy of $^{13}C\{^{1}H\}$ (100.61 MHz, $CDCl_3$) NMR spectrum of **4e**

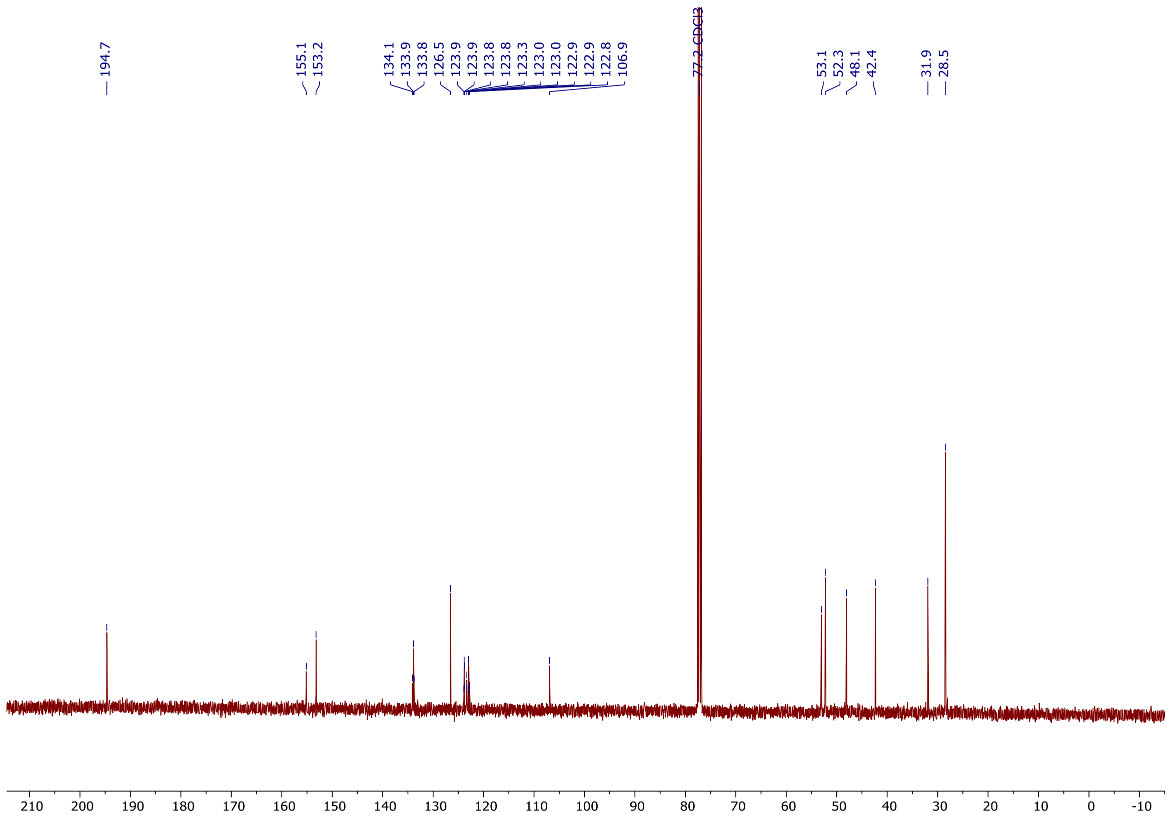

Copy of $^{19}F\{^{1}H\}$ (376.50 MHz, $CDCl_3$) NMR spectrum of **4e**

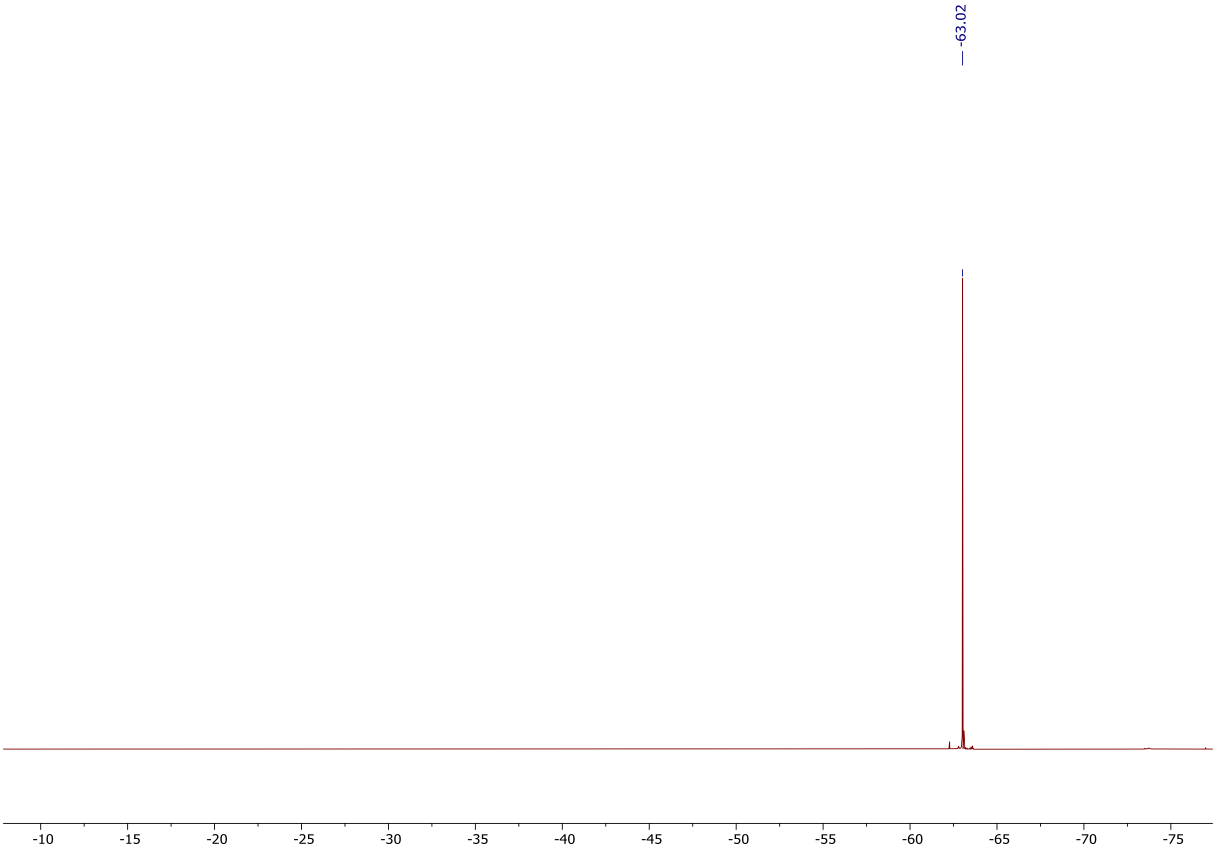

Copy of ^{1}H (400.13 MHz, CDCl$_3$) ^{19}F{^{1}H} (376.50 MHz, CDCl$_3$) NMR spectrum of **4f**

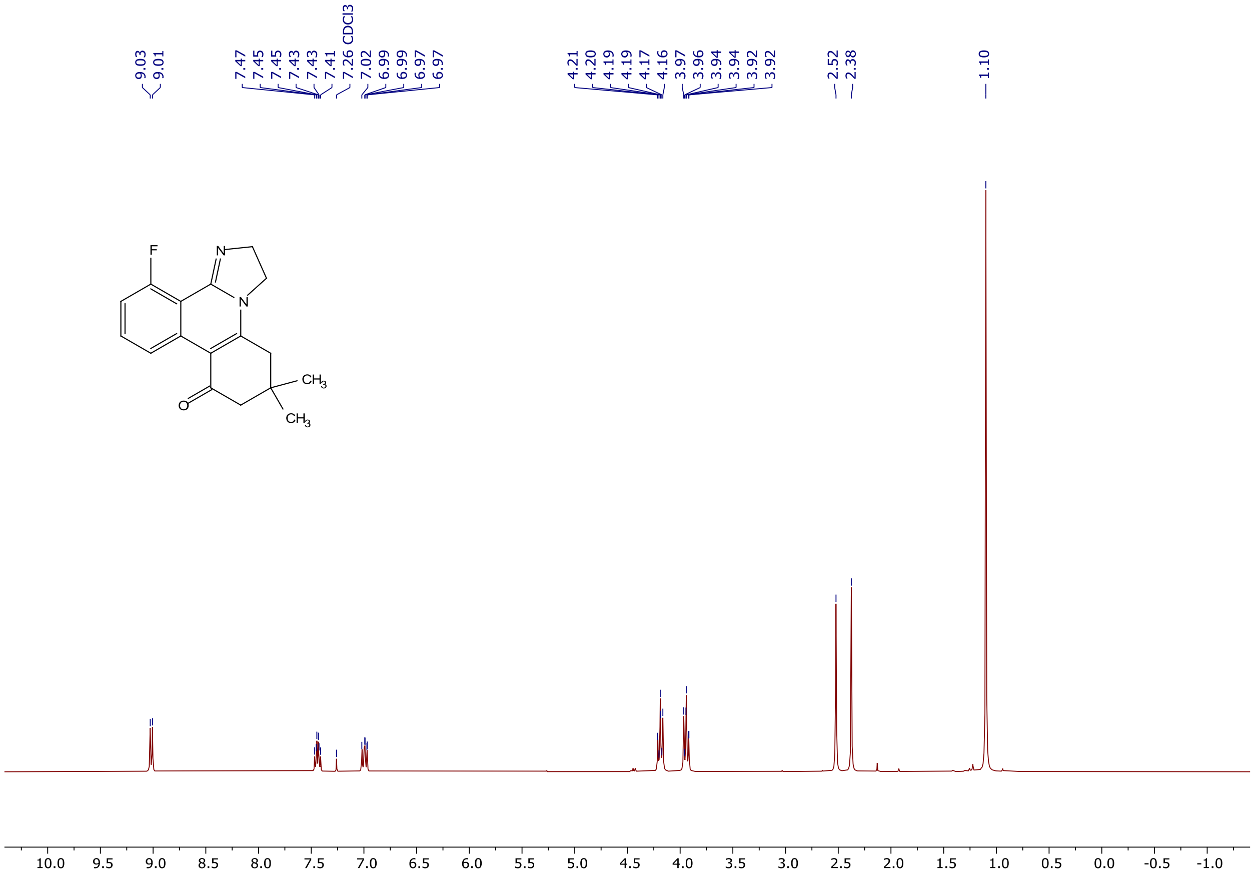

Copy of ^{13}C{^{1}H} (100.61 MHz, CDCl$_3$) NMR spectrum of **4f**

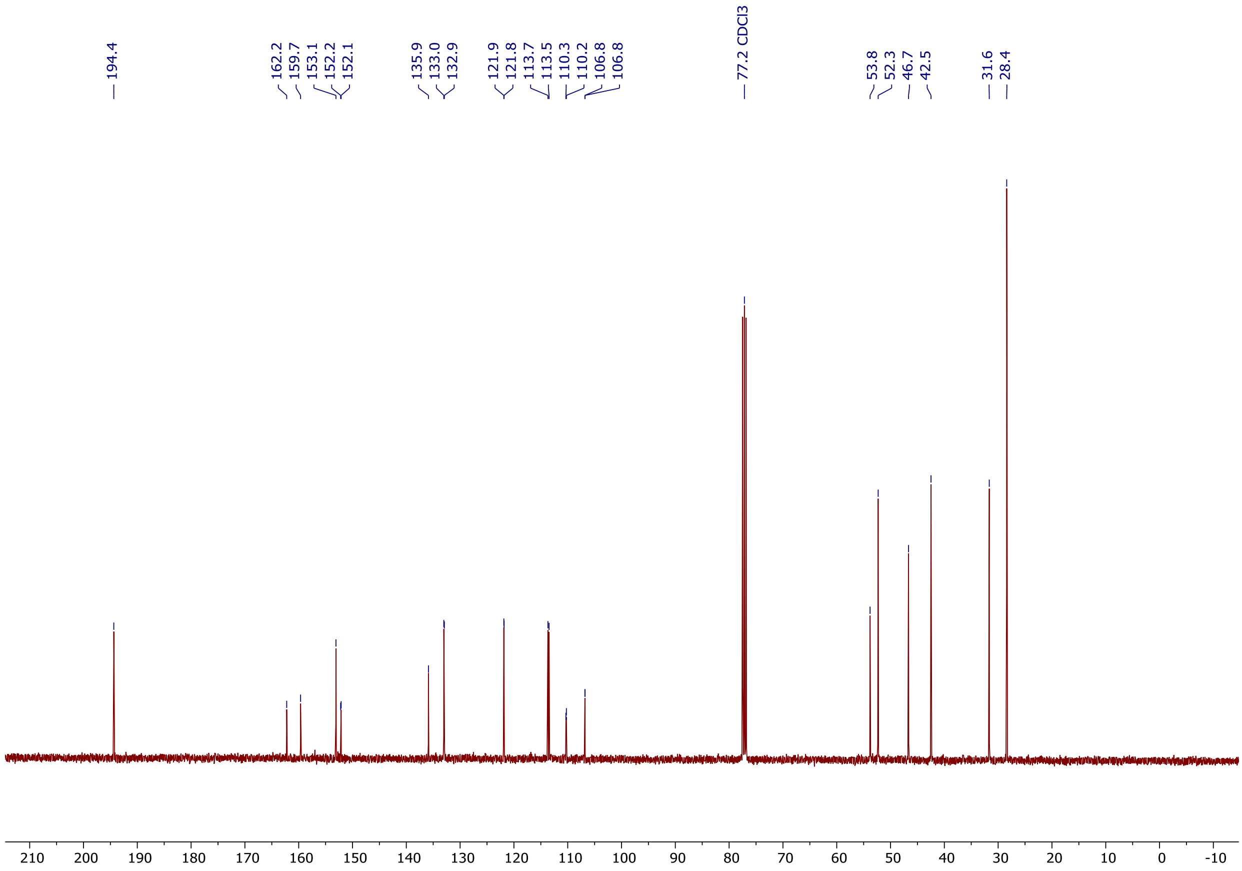

Copy of $^{19}F\{^1H\}$ (376.50 MHz, $CDCl_3$) NMR spectrum of **4f**

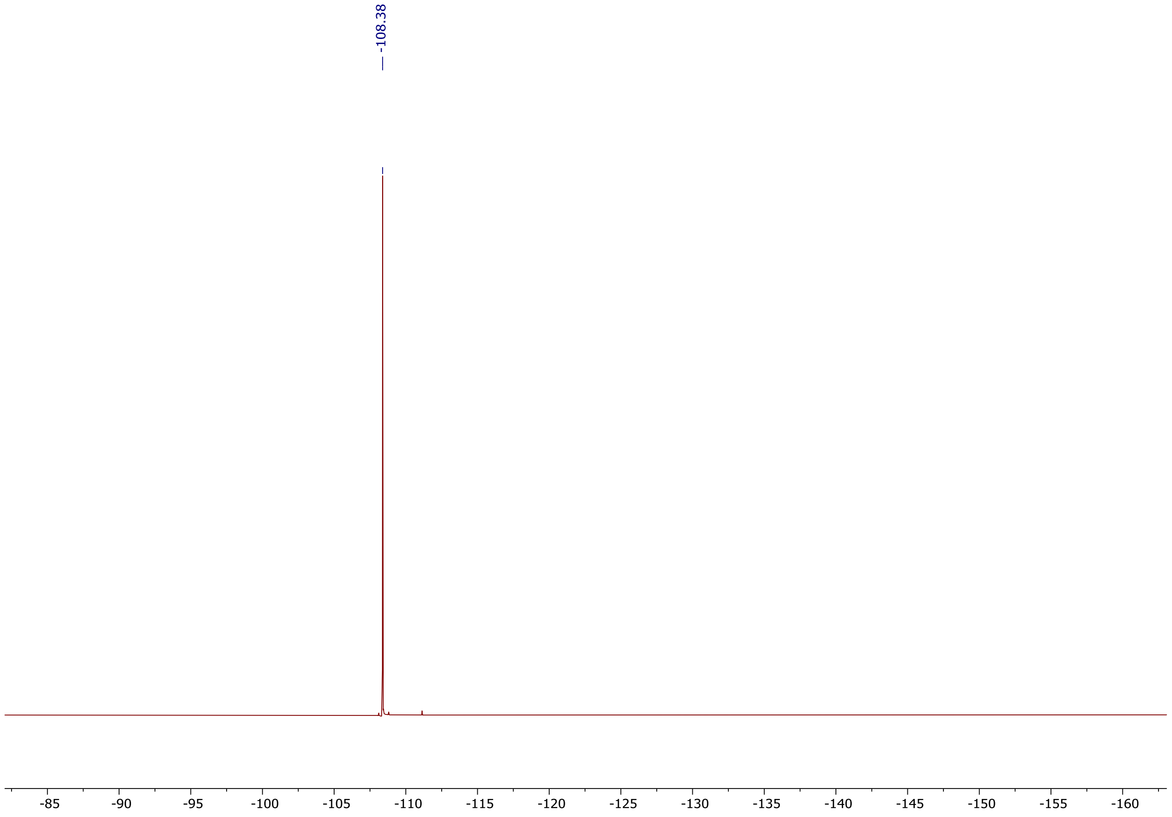

Copy of ^{1}H (400.13 MHz, $CDCl_3$) NMR spectrum of **4g + g’**

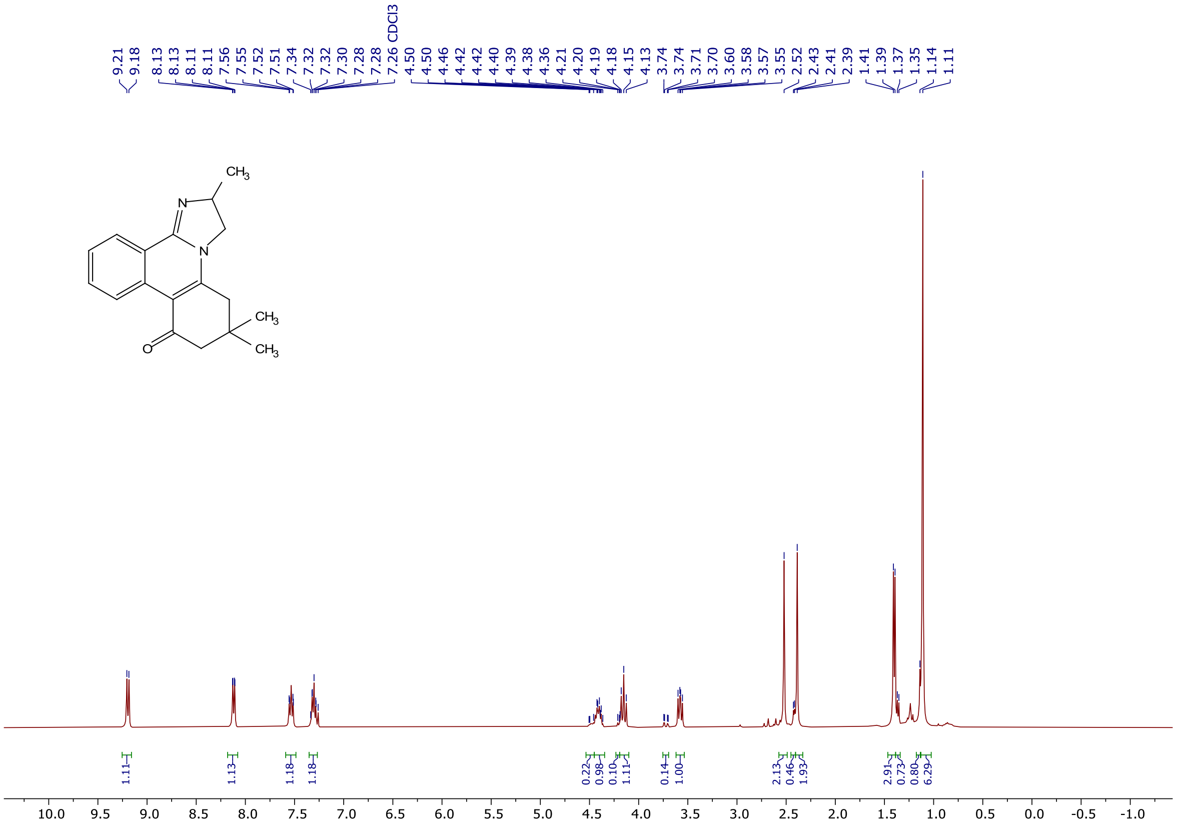

Copy of $^{13}C\{^{1}H\}$ (100.61 MHz, $CDCl_3$) NMR spectrum of **4g + g’**

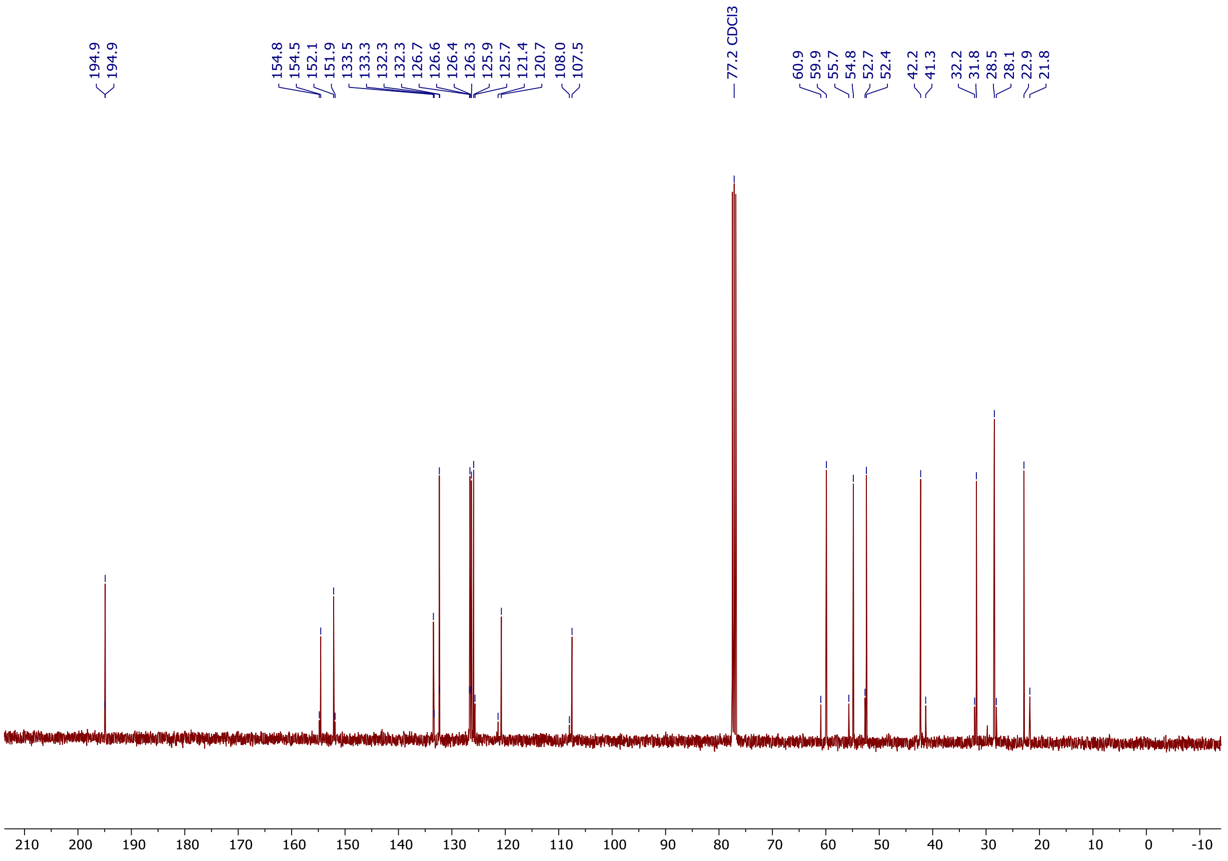

Copy of NOESY (400.13 MHz, $CDCl_3$) NMR spectrum of **4g + g'**

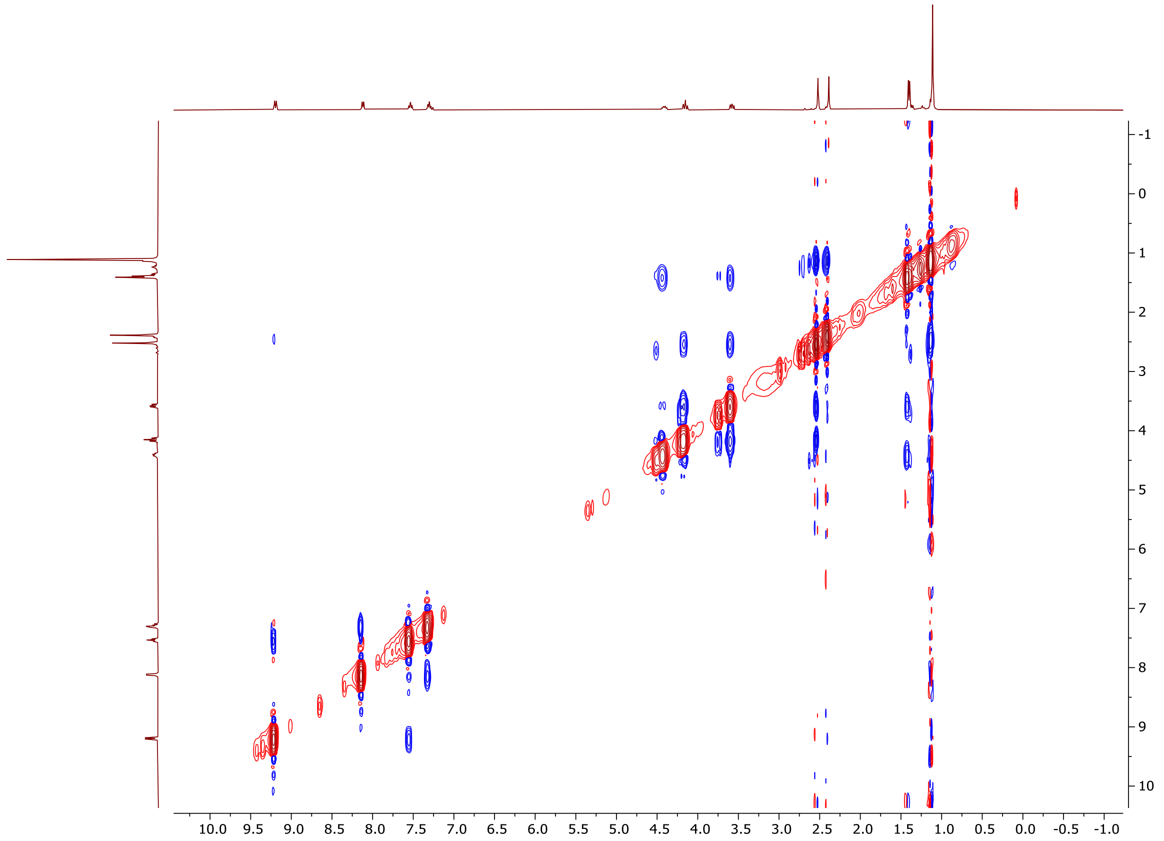

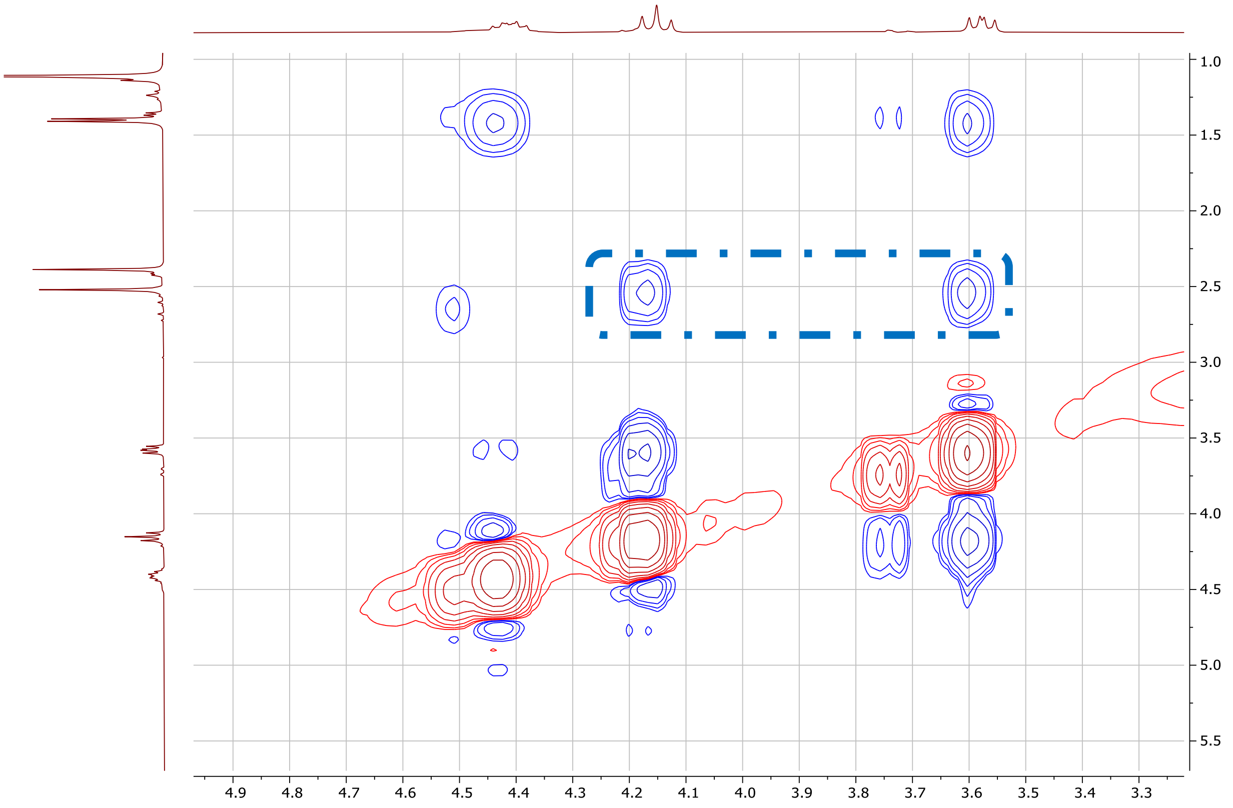

Copy of ^{1}H (400.13 MHz, $CDCl_3$) NMR spectrum of **4h**

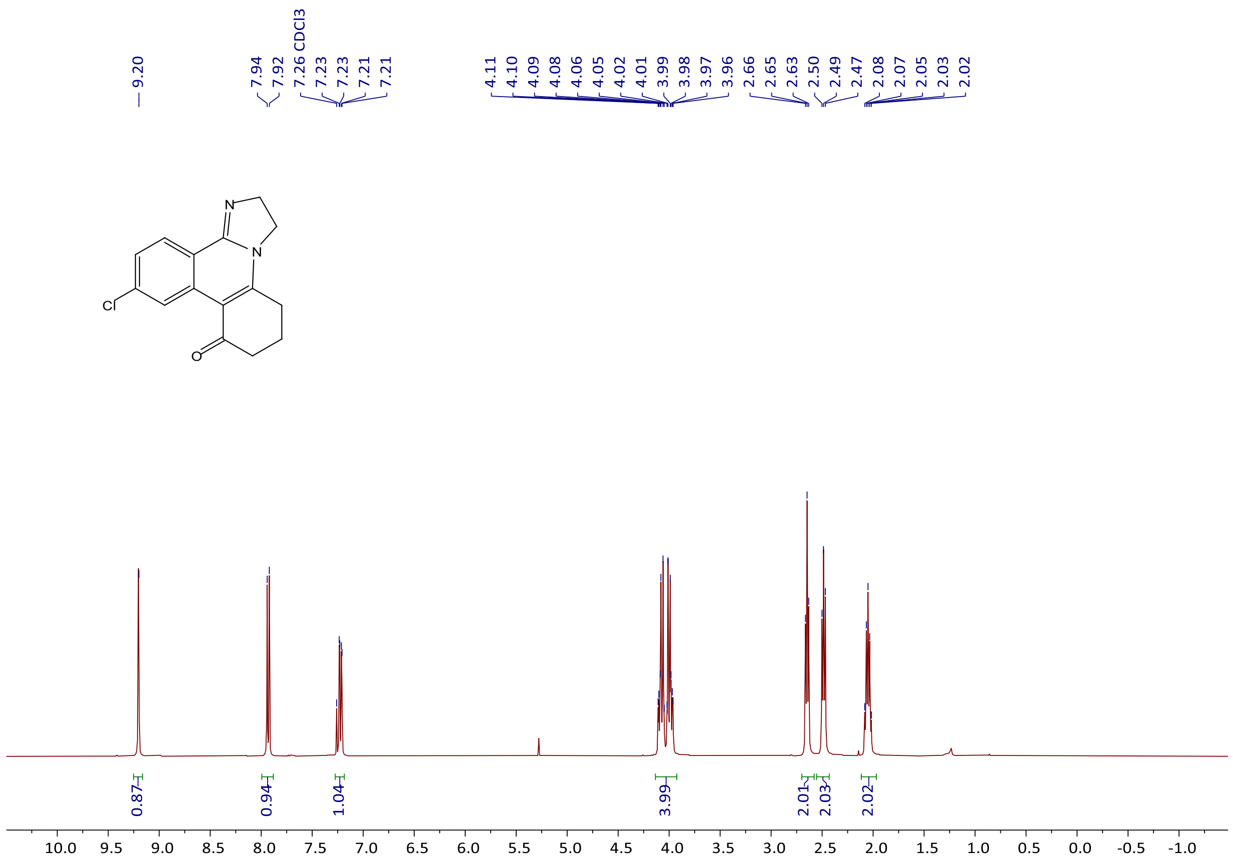

Copy of $^{13}C\{^{1}H\}$ (100.61 MHz, $CDCl_3$) NMR spectrum of **4h**

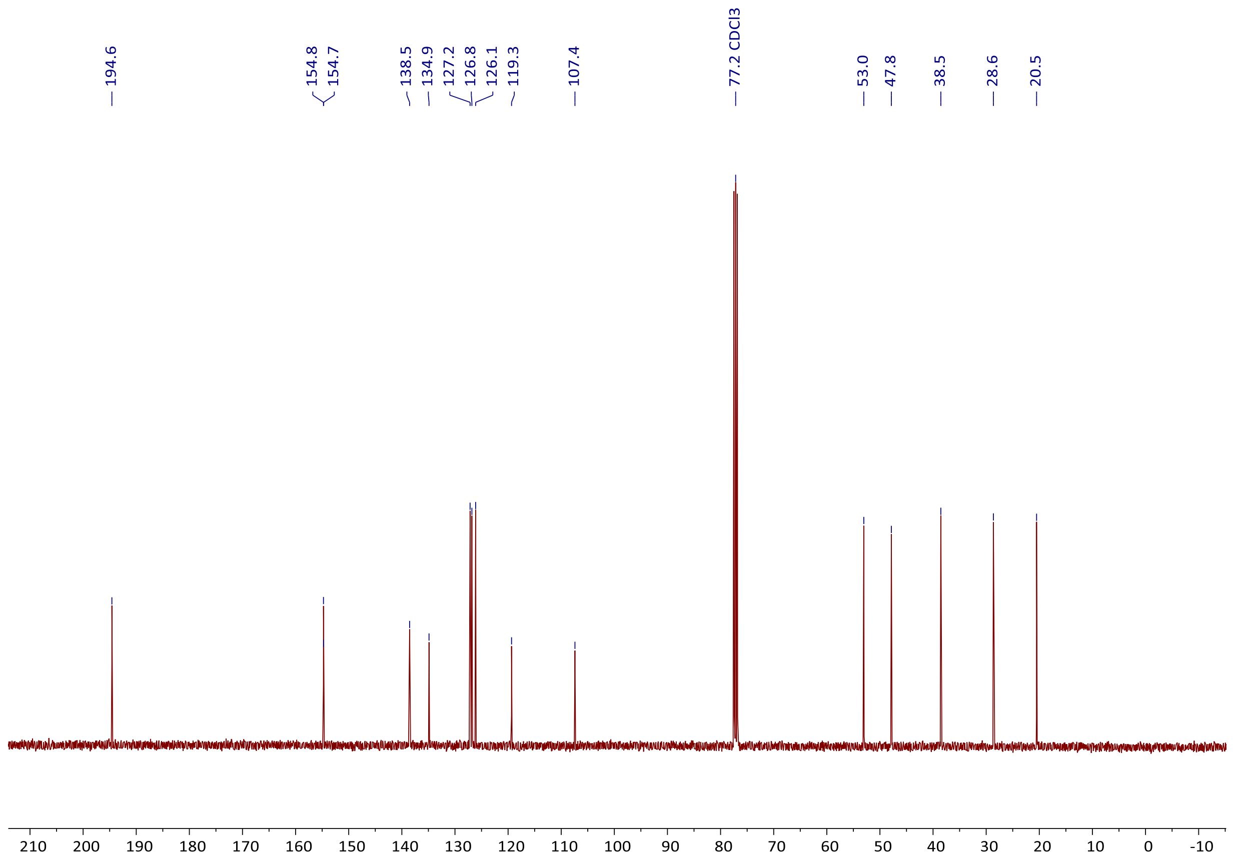

Copy of ^{1}H (400.13 MHz, $CDCl_3$) NMR spectrum of **4i**

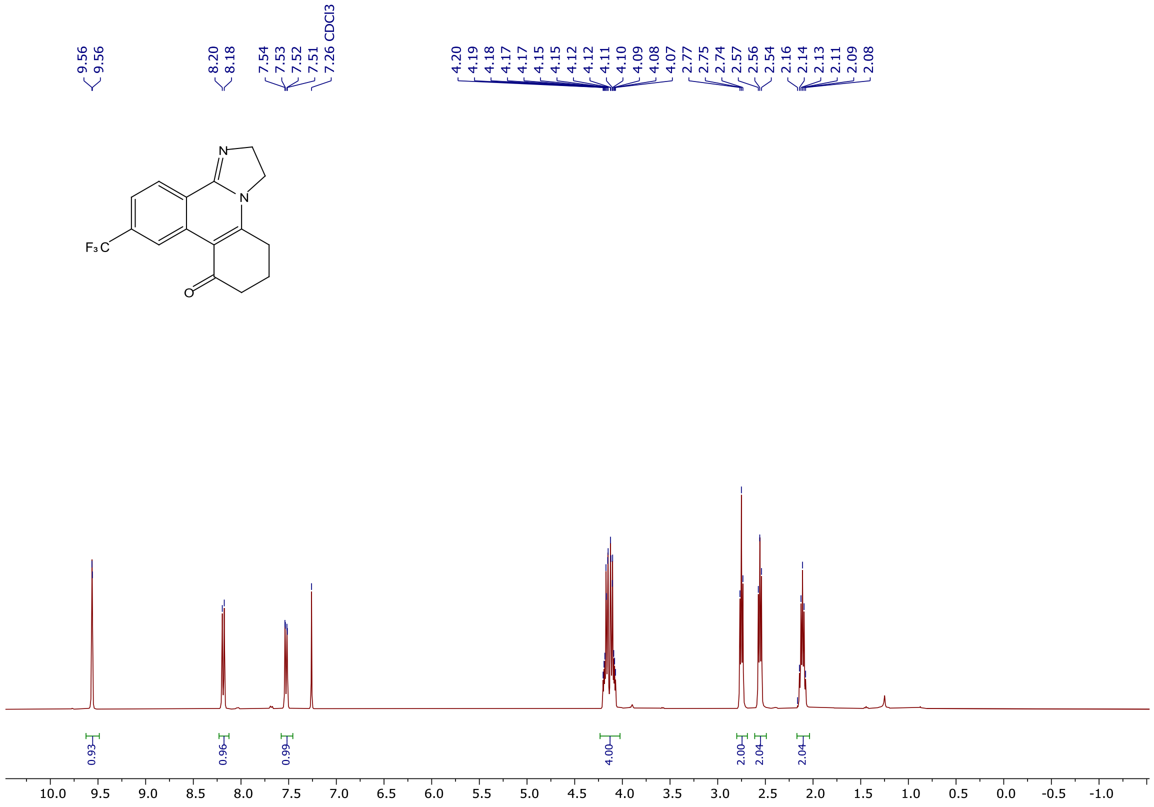

Copy of $^{13}C\{^{1}H\}$ (100.61 MHz, $CDCl_3$) vspectrum of **4i**

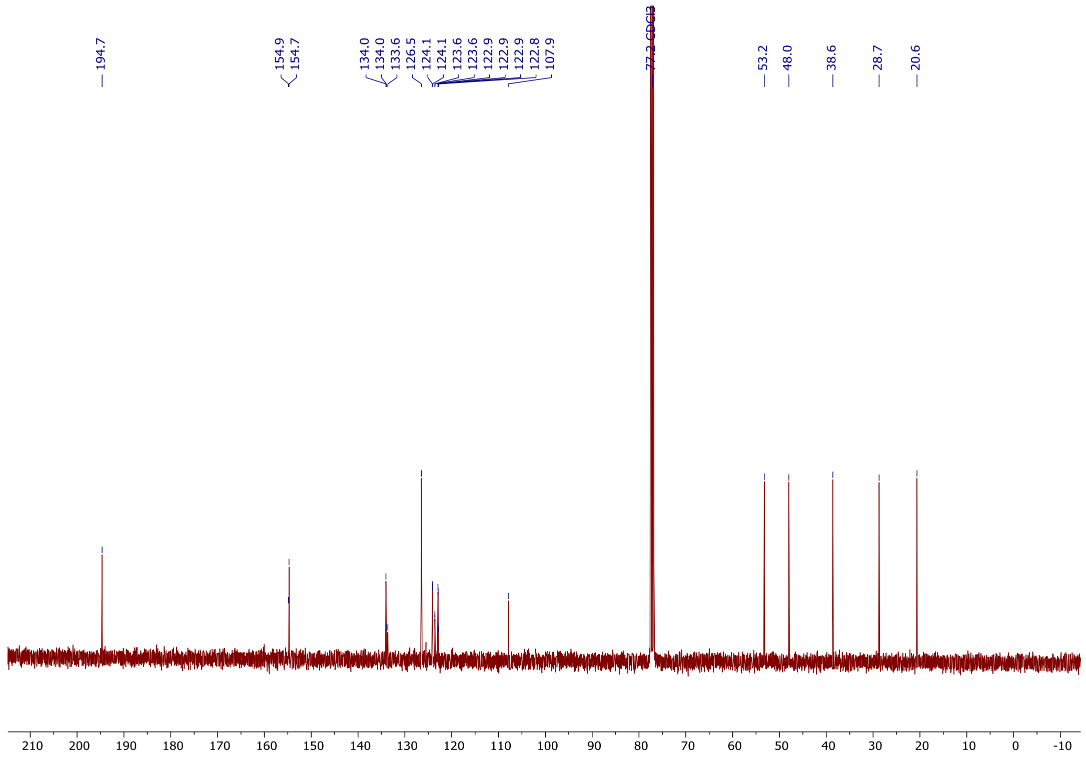

Copy of $^{19}F\{^{1}H\}$ (376.50 MHz, $CDCl_3$) NMR spectrum of **4i**

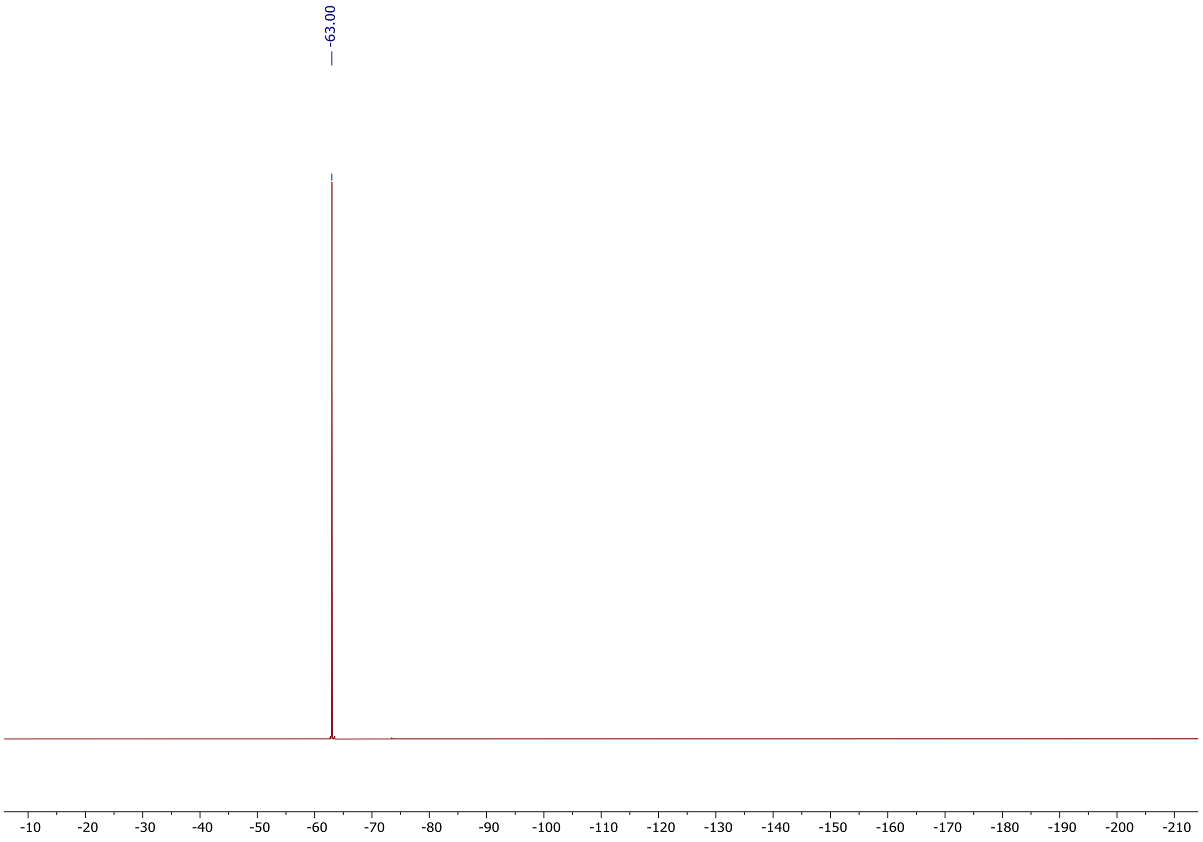

Copy of ^{1}H (400.13 MHz, $CDCl_3$) NMR spectrum of **4j**

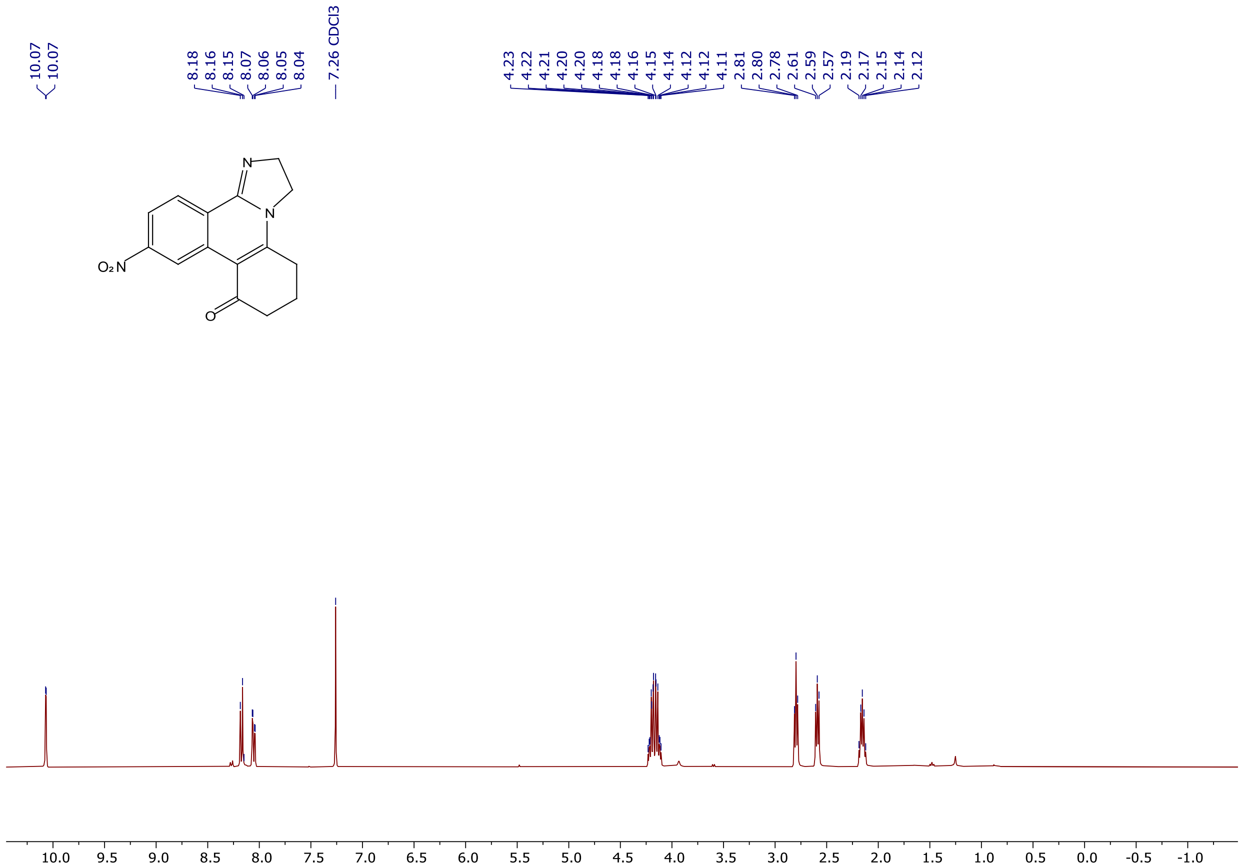

Copy of $^{13}C\{^{1}H\}$ (100.61 MHz, $CDCl_3$) NMR spectrum of **4j**

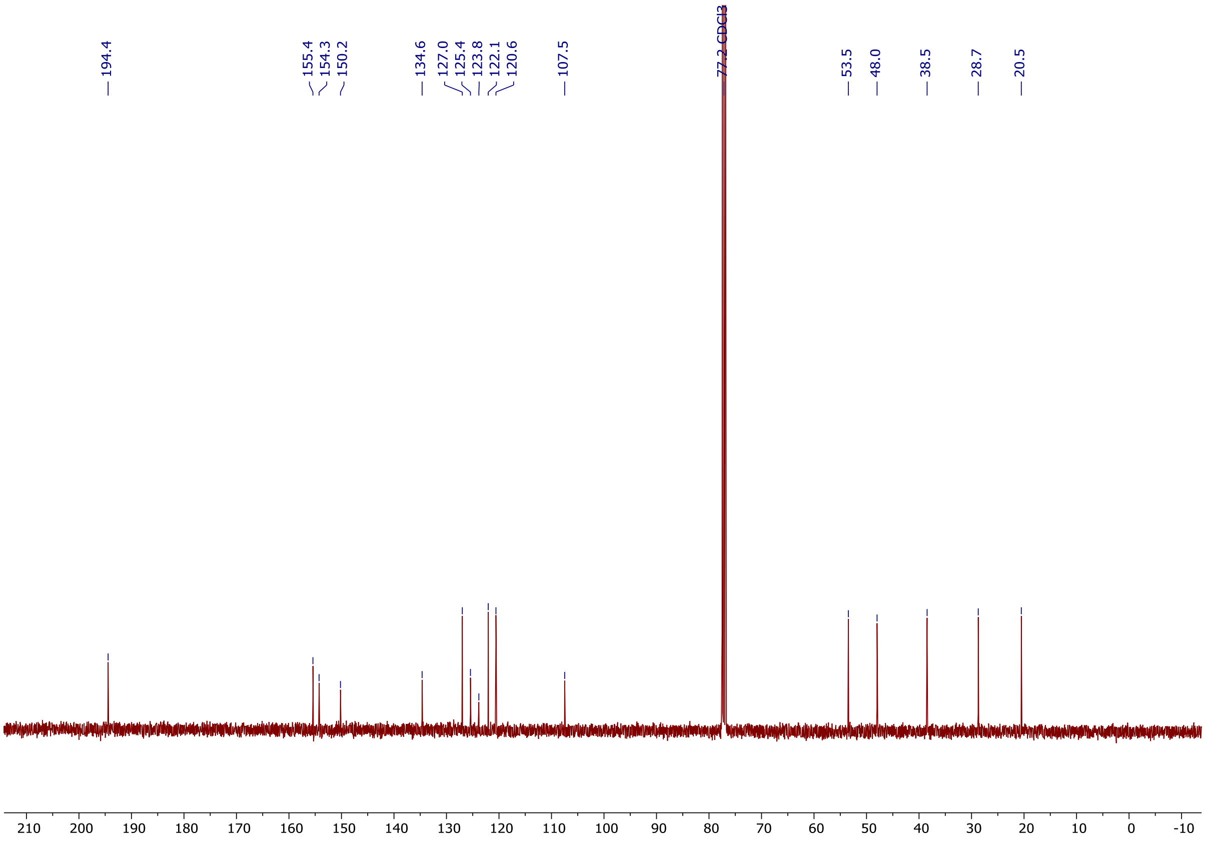

Copy of ^{1}H (400.13 MHz, $CDCl_3$) NMR spectrum of **4k**

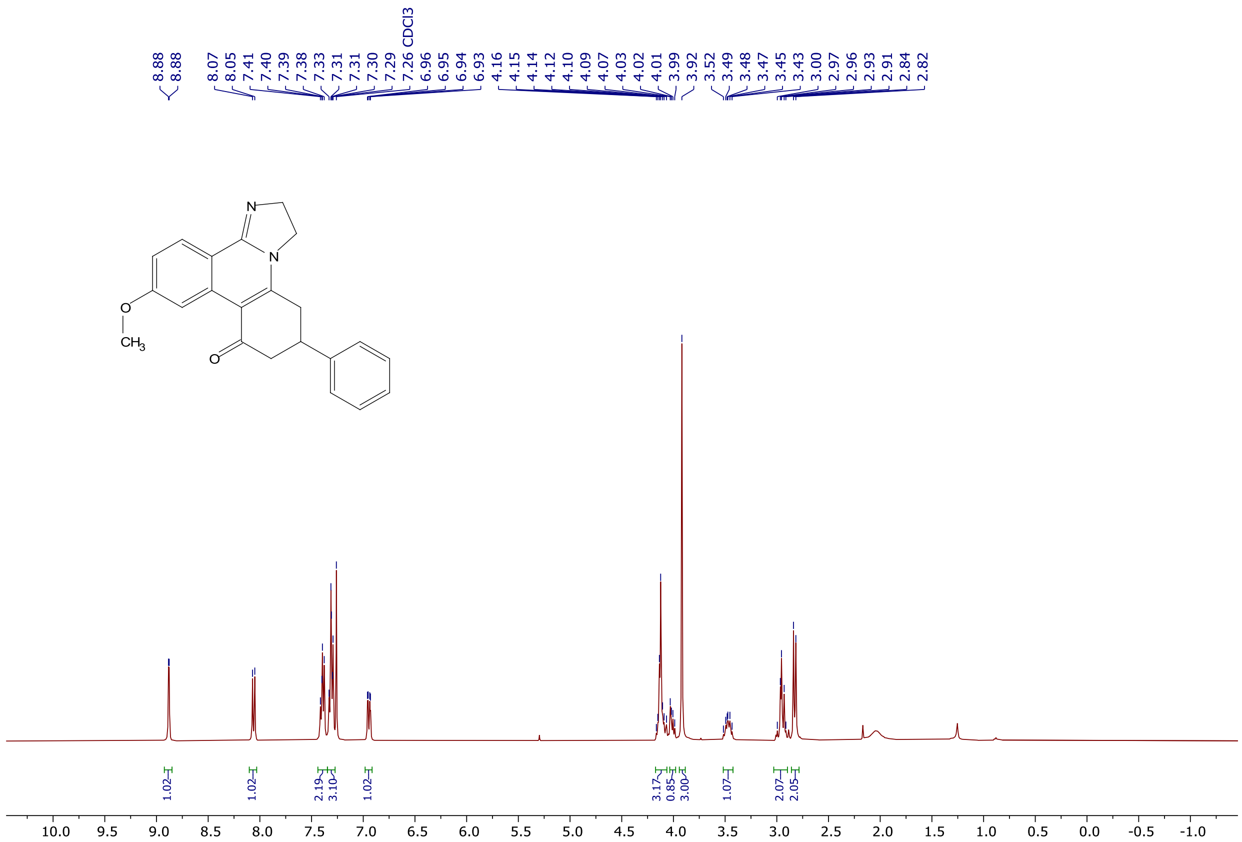

Copy of $^{13}C\{^{1}H\}$ (100.61 MHz, $CDCl_3$) NMR spectrum of **4k**

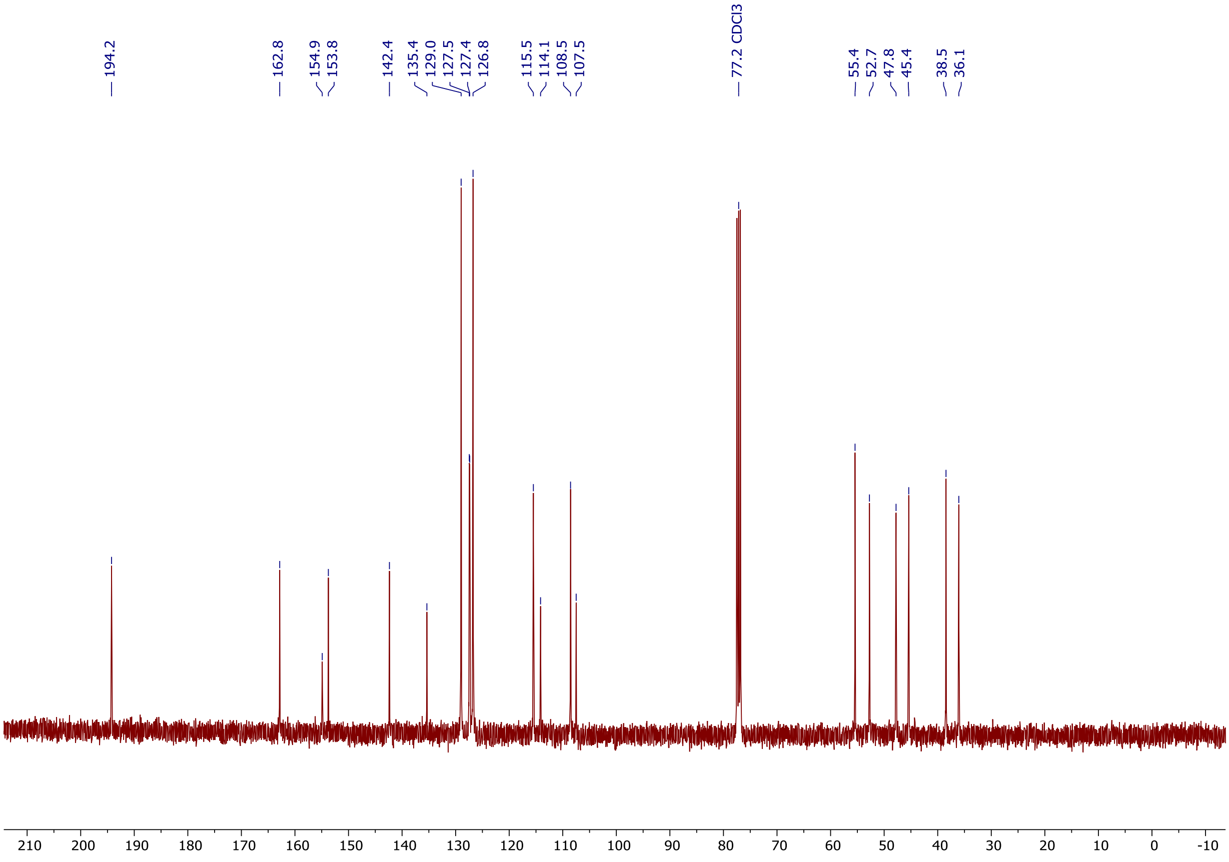

Copy of ^{1}H (400.13 MHz, $CDCl_3$) NMR spectrum of **4l**

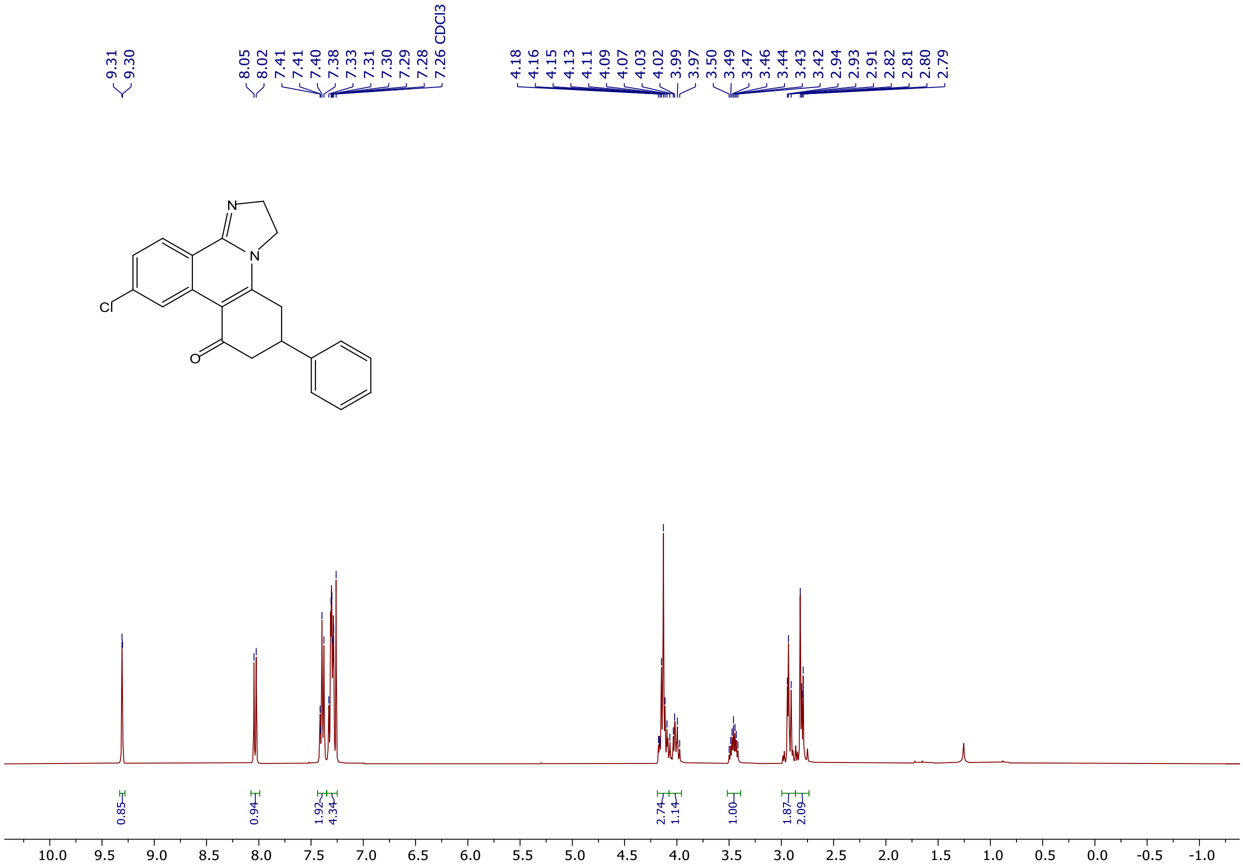

Copy of ^{13}C{^{1}H} (100.61 MHz, $CDCl_3$) NMR spectrum of **4l**

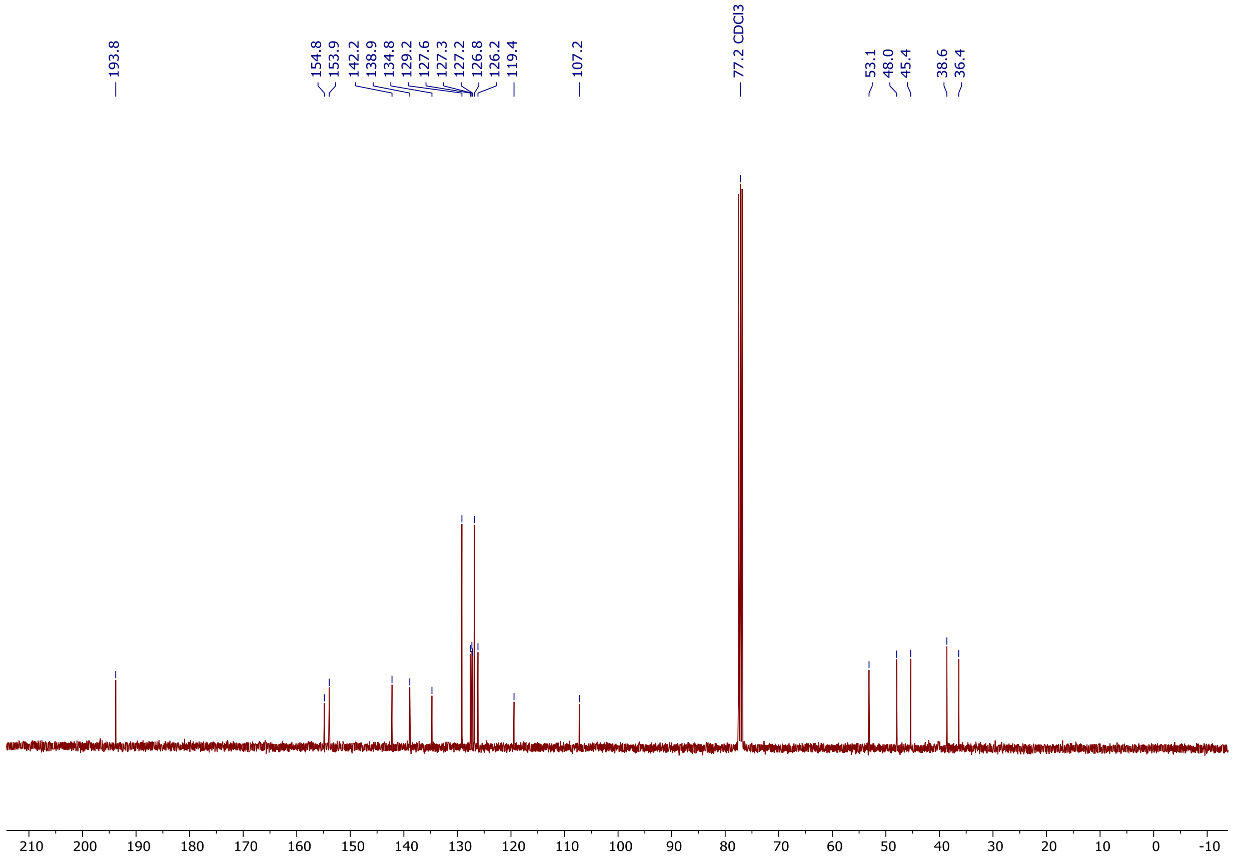

Copy of ^{1}H (400.13 MHz, $CDCl_3$) NMR spectrum of **4m**

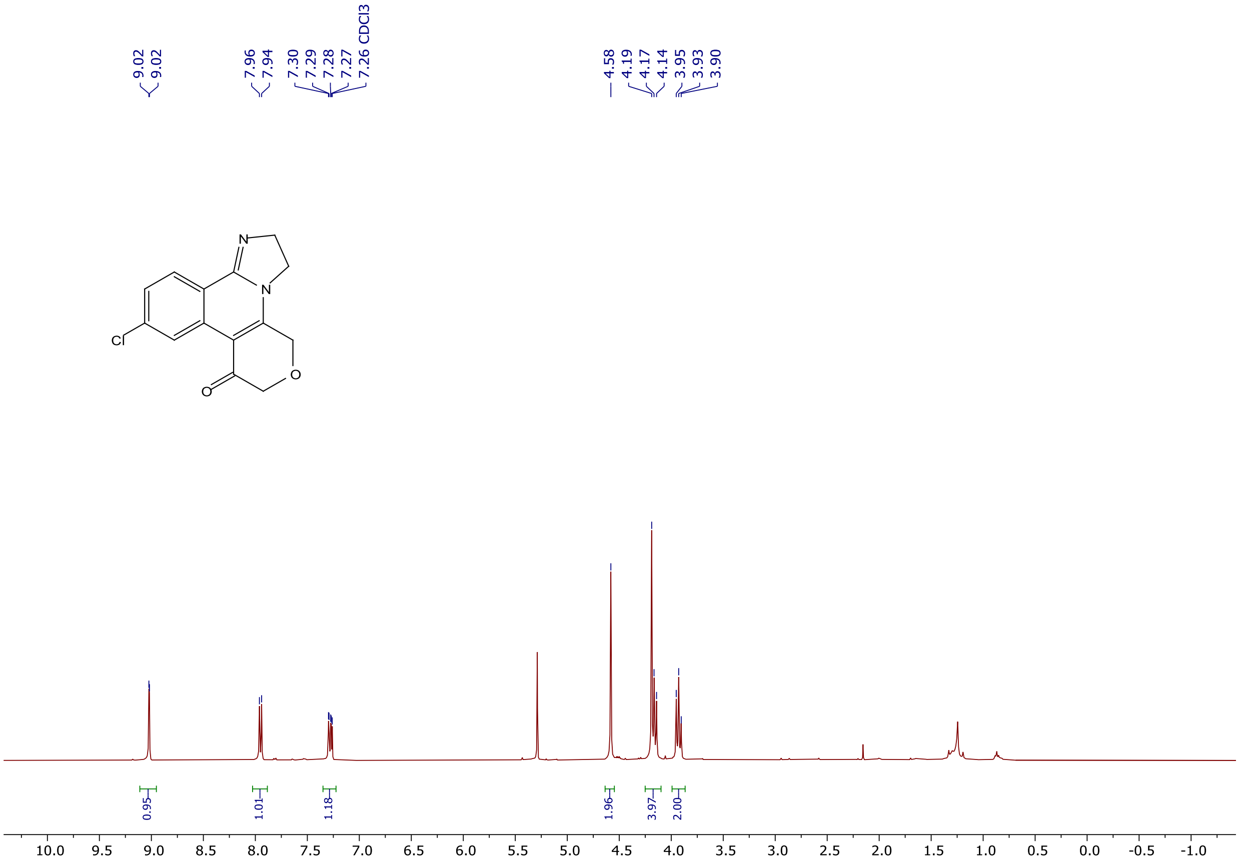

Copy of $^{13}C\{^{1}H\}$ (100.61 MHz, $CDCl_3$) NMR spectrum of **4m**

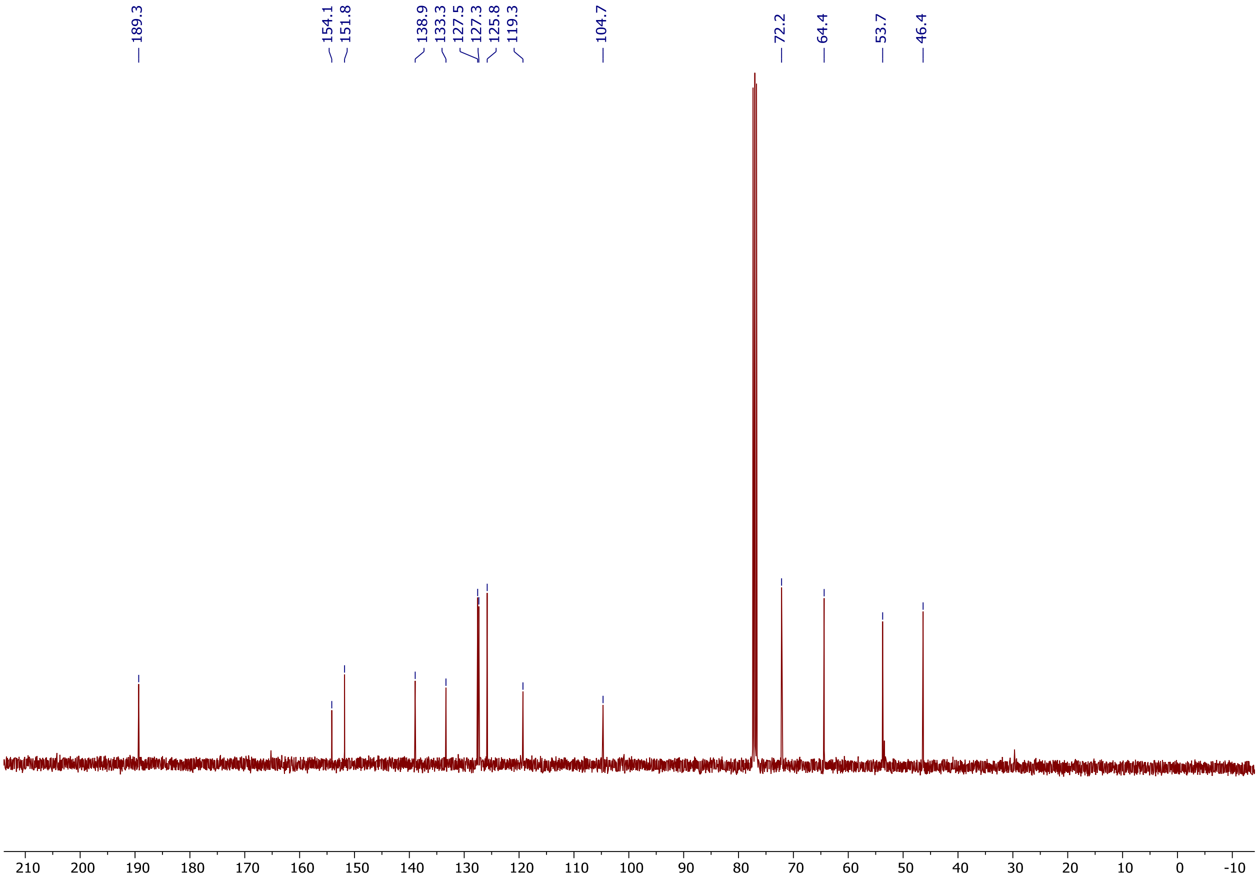

Copy of ^{1}H (400.13 MHz, $CDCl_3$) and $^{13}C\{^{1}H\}$ (100.61 MHz, $CDCl_3$) NMR spectrum of **4n**

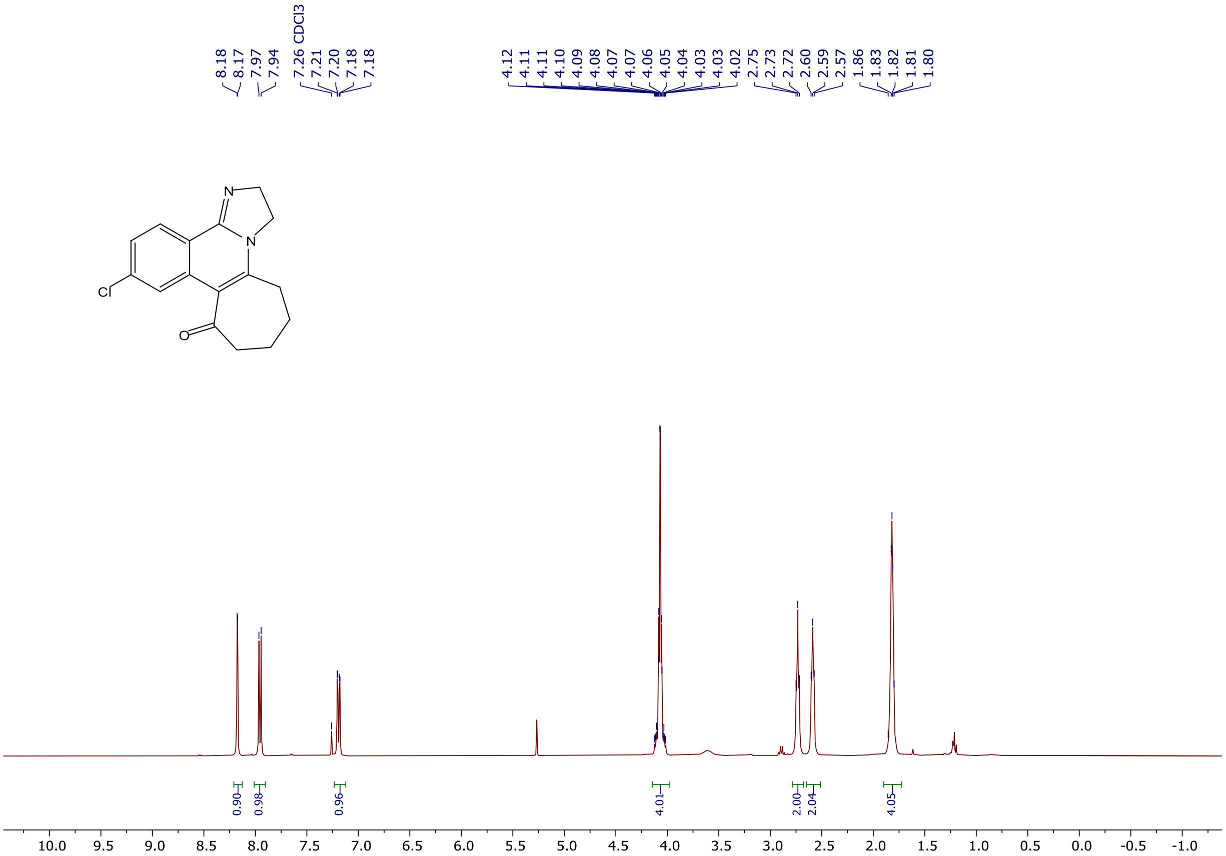

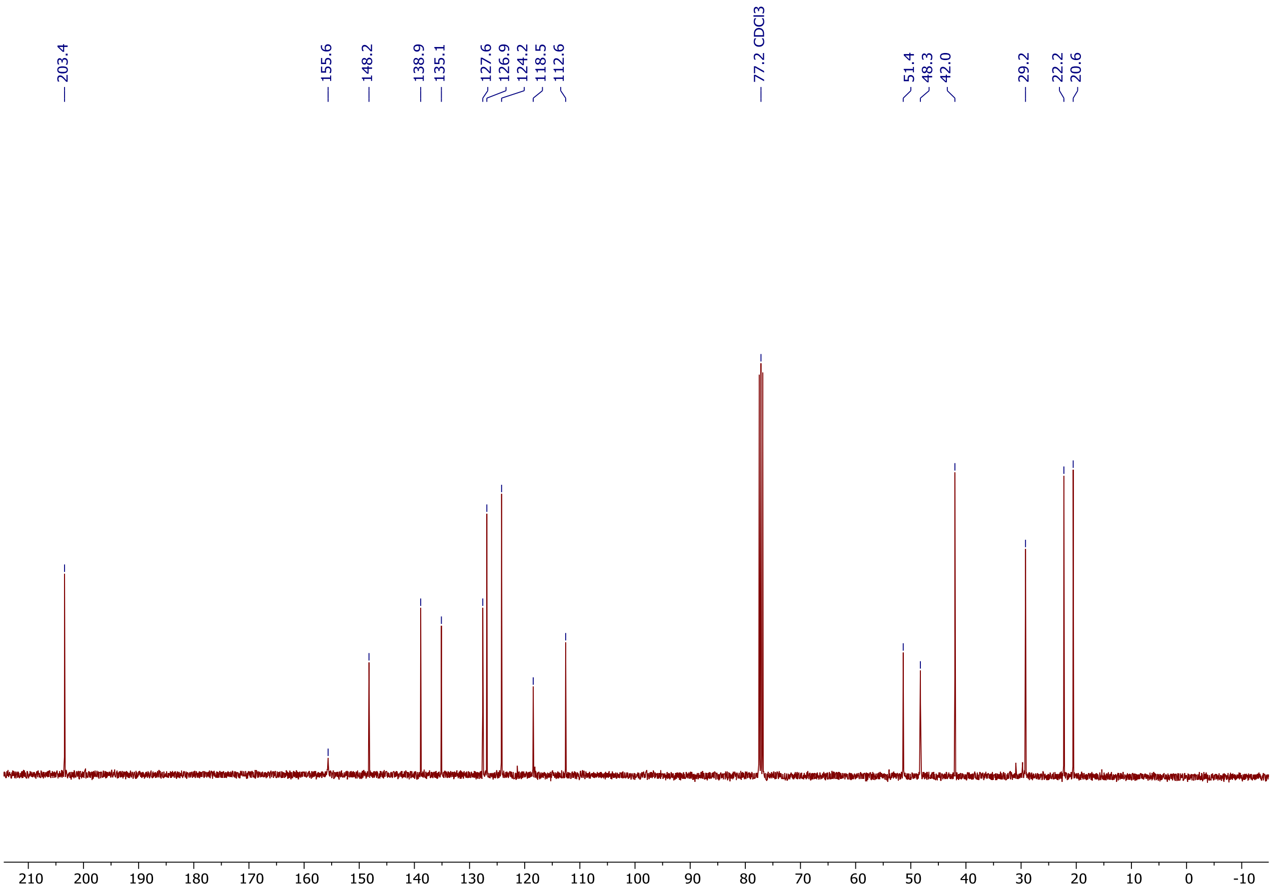

Copy of ^{1}H (400.13 MHz, DMSO-d_6+$CDCl_3$ in a ratio 1:6) NMR spectrum of **4o**

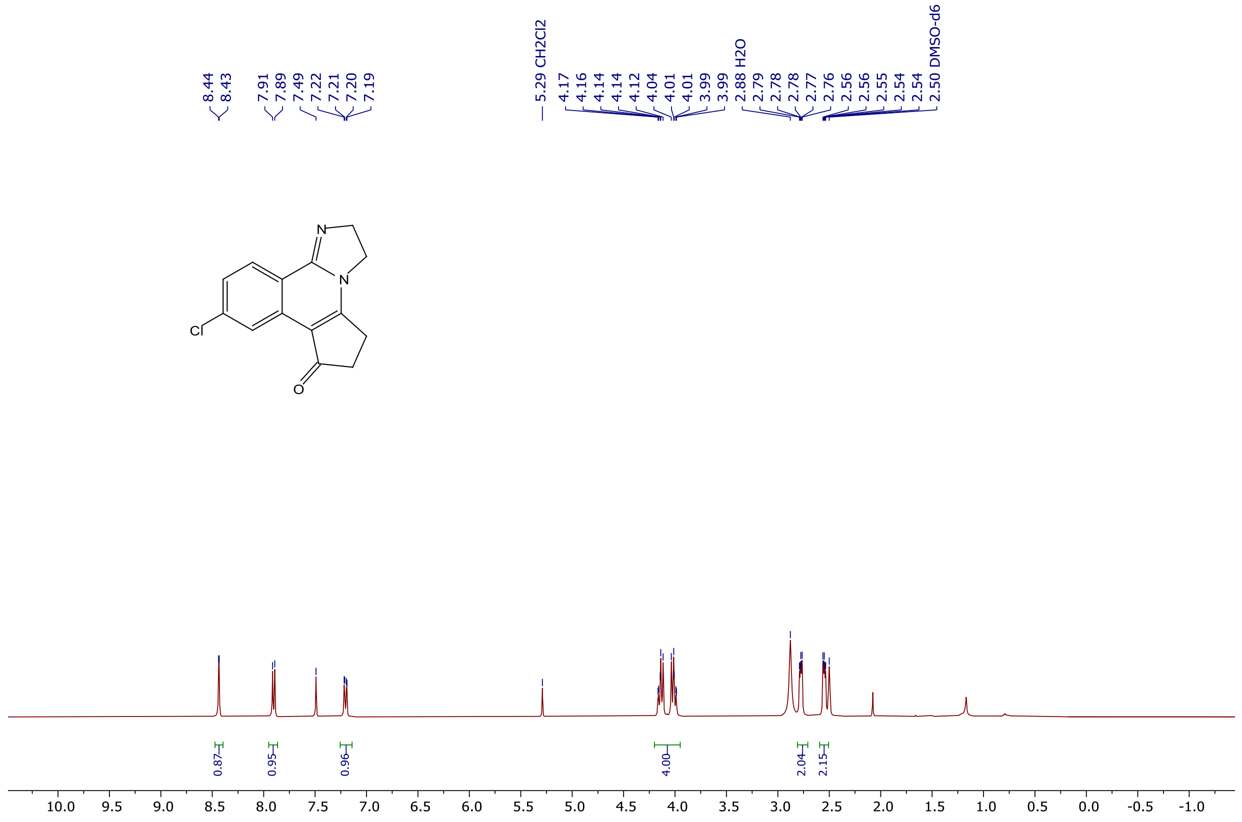

Copy of ^{13}C{^{1}H} (100.61 MHz, DMSO-d_6+$CDCl_3$ in a ratio 1:6) NMR spectrum of **4o**

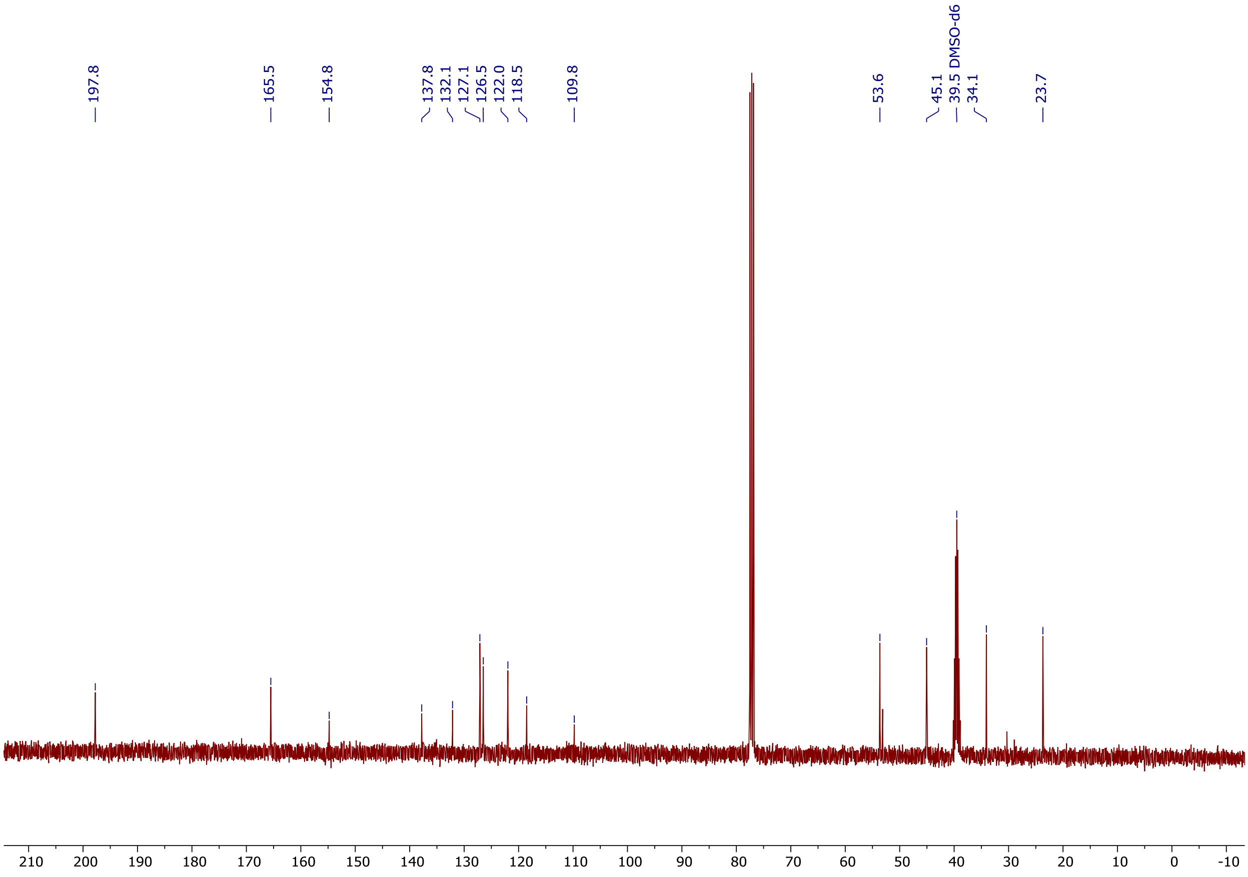

Copy of ^{1}H (400.13 MHz, $CDCl_3$) NMR spectrum of **4p**

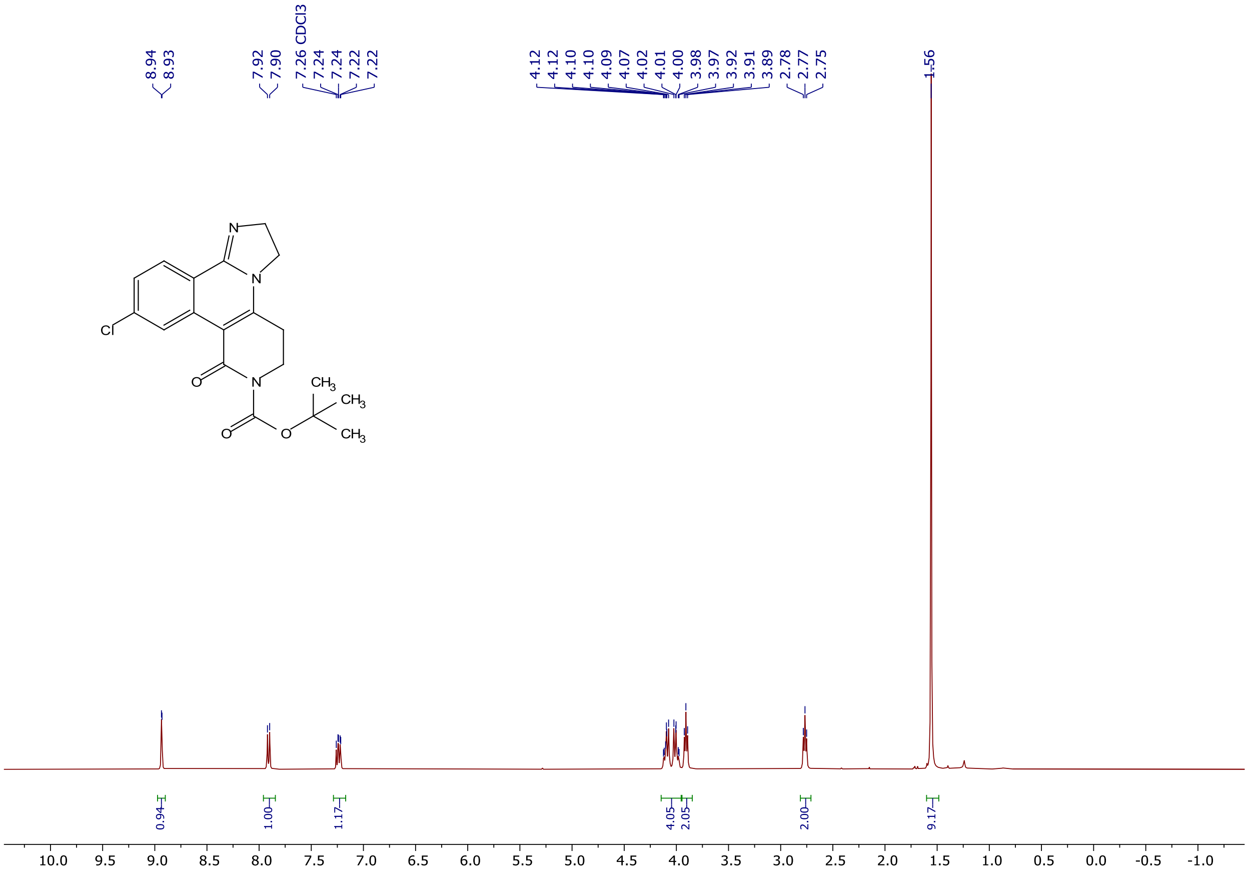

Copy of $^{13}C\{^{1}H\}$ (100.61 MHz, $CDCl_3$) NMR spectrum of **4p**

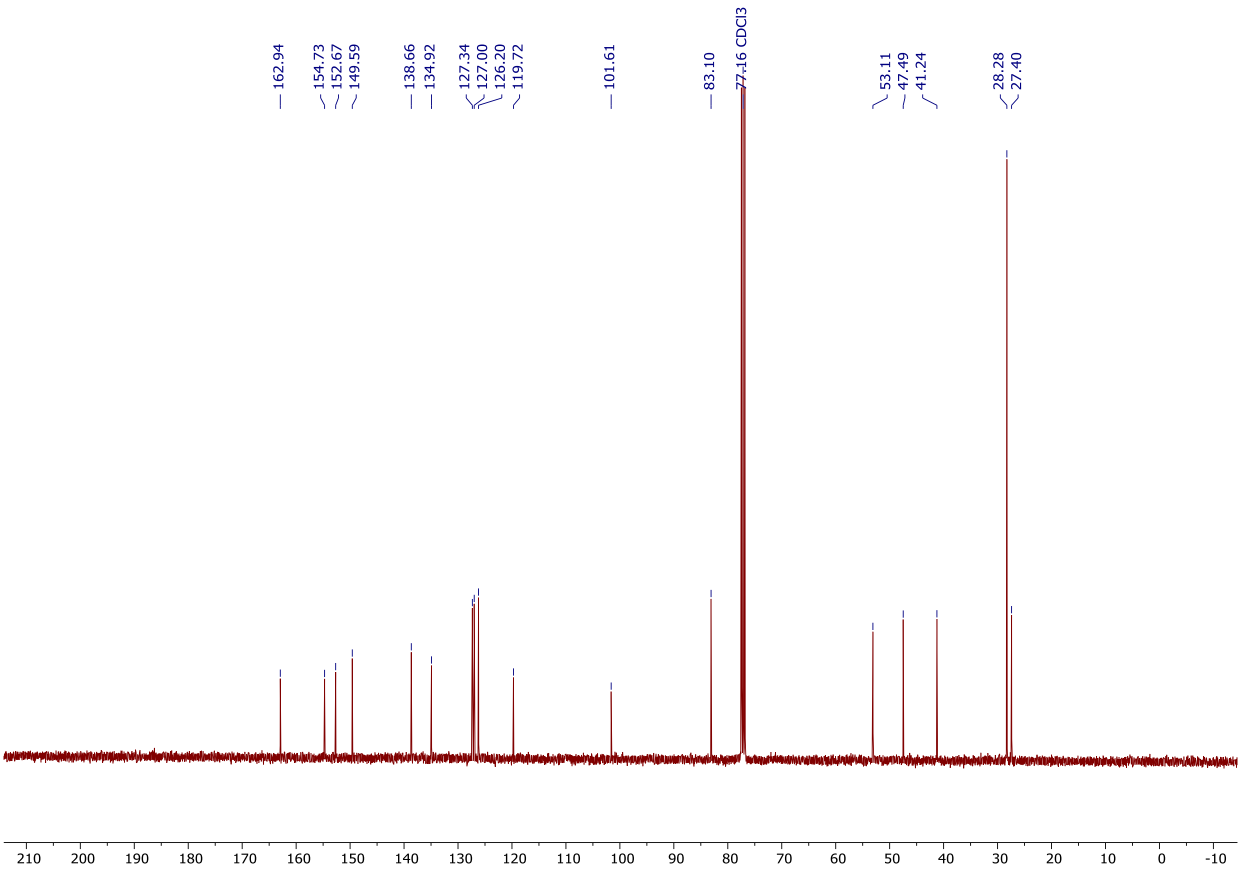

Copy of ^{1}H (400.13 MHz, $CDCl_3$) NMR spectrum of **4q**

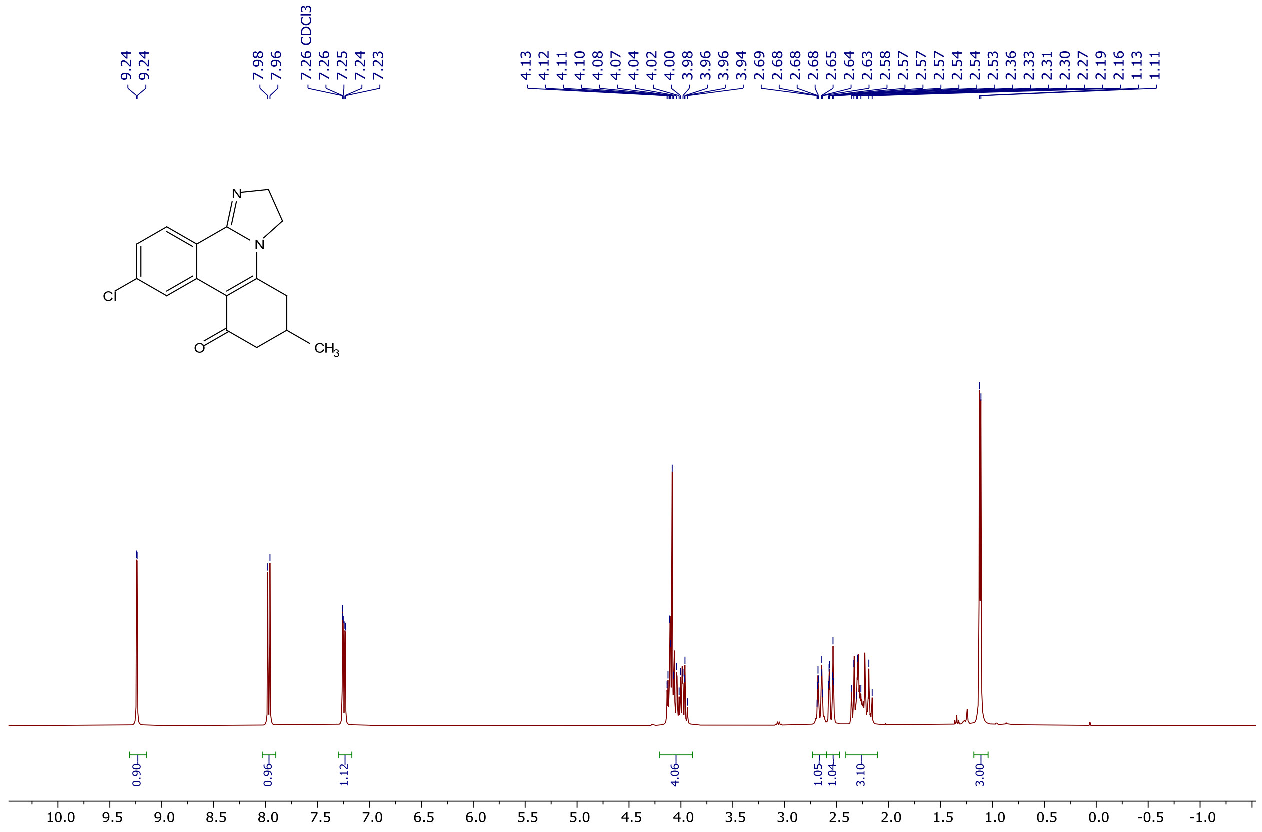

Copy of $^{13}C\{^{1}H\}$ (100.61 MHz, $CDCl_3$) NMR spectrum of **4q**

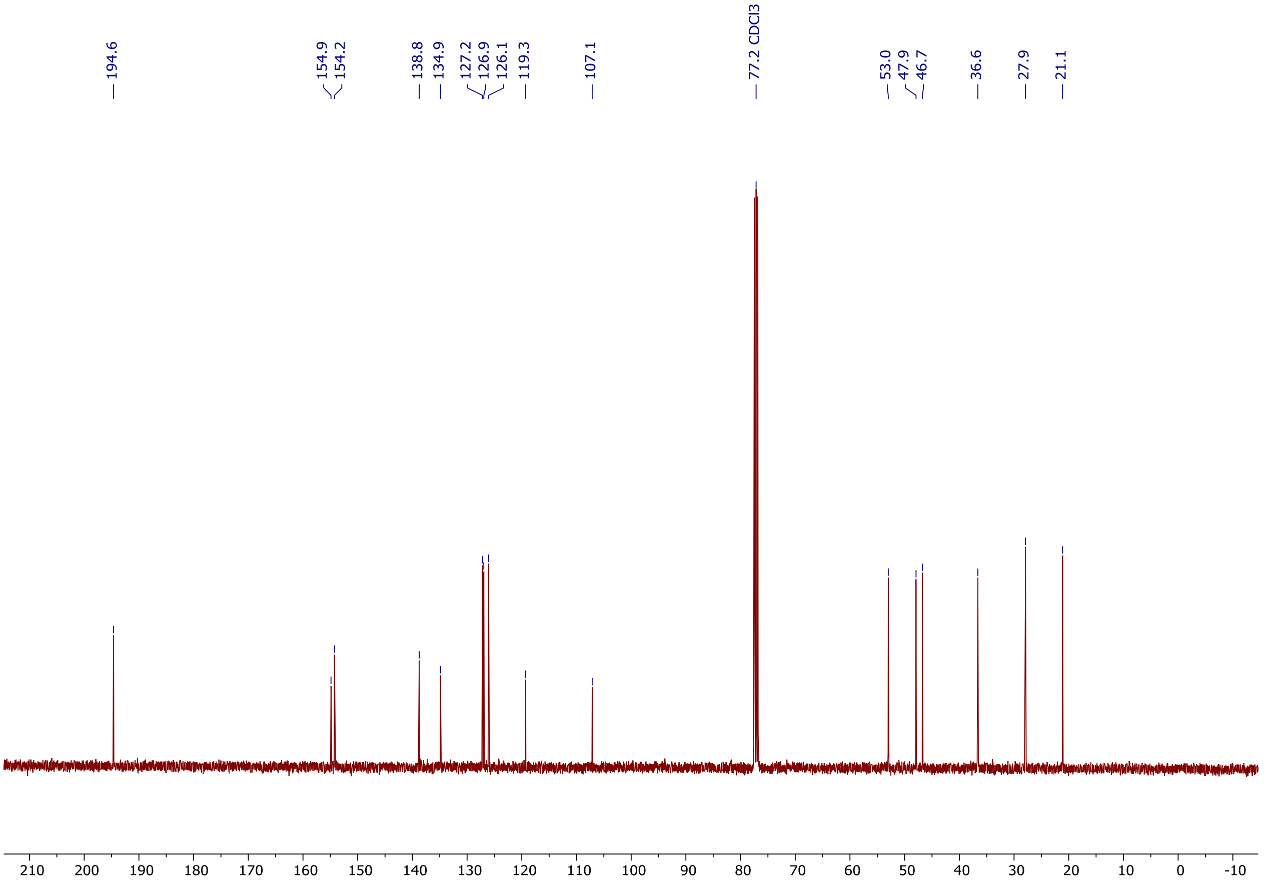

3. Crystallographic data for compounds 4a and 4f

X-ray single crystal analysis was performed on a SuperNova diffractometer. Crystals were kept at 100(2) K during data collection. Using Olex2[4], the structures were solved with the SHELXT[5] structure solution program using intrinsic phasing and refined with the SHELXL[6] refinement package using least squares minimisation.

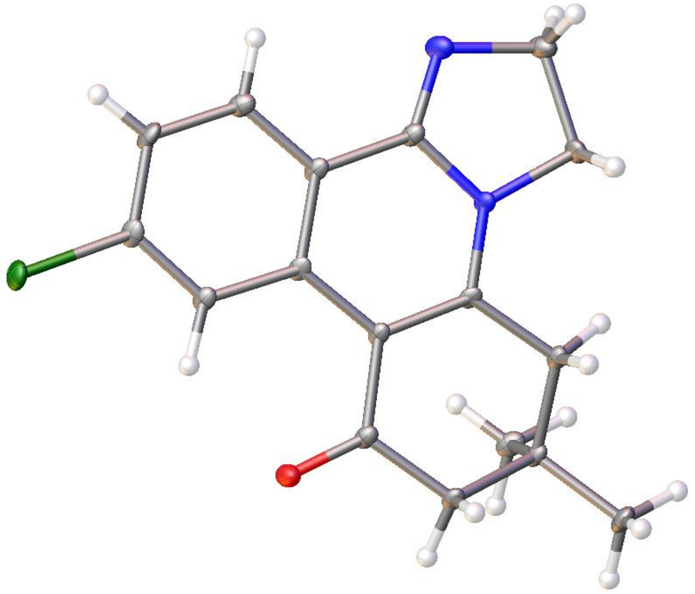

Figure S2. ORTEP representation of compound **4a** (thermal ellipsoids are shown at 50% probability).

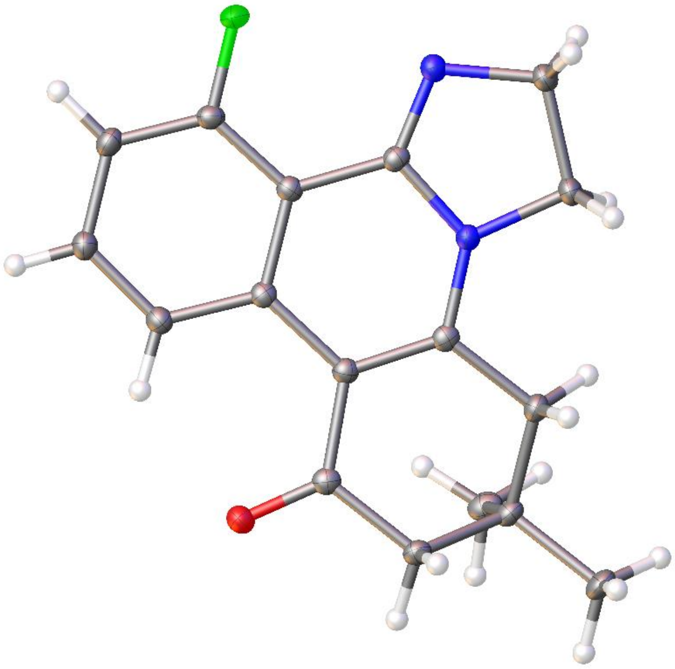

Figure S3. ORTEP representation of compound **4f** (thermal ellipsoids are shown at 50% probability).

**Table S1.** Crystal data and structure refinement for **4a** and **4f.**		
**Compound number**	**4a**	**4f**
**CCDC**	**2450071**	**2502221**
Empirical formula	$C_{17}H_{17}ClN_2O$	$C_{17}H_{17}FN_2O$
Formula weight	300.77	284.32
Temperature/K	99.9(2)	100.15
Crystal system	monoclinic	monoclinic
Space group	$P2_1/c$	$P2_1/c$
a/Å	6.2845(2)	8.24950(10)
b/Å	13.6733(3)	13.9108(2)
c/Å	16.7189(4)	12.9245(2)
α/°	90	90
β/°	91.028(2)	106.252(2)
γ/°	90	90
Volume/Å^3	1436.42(7)	1423.91(4)
Z	4	4
ρ_{calc} g/cm^3	1.391	1.326
μ/mm^{-1}	2.347	0.755
F(000)	632.0	600.0
Crystal size/mm^3	0.22 × 0.16 × 0.12	0.18 × 0.14 × 0.12
Radiation	Cu Kα (λ = 1.54184)	CuKα (λ = 1.54184)
2Θ range for data collection/°	8.354 to 147.342	9.552 to 158.838
Index ranges	-6 ≤ h ≤ 7, -16 ≤ k ≤ 9, -20 ≤ l ≤ 18	-10 ≤ h ≤ 9, -14 ≤ k ≤ 17, -13 ≤ l ≤ 16
Reflections collected	5289	10502

Independent reflections	2804 [R_{int} = 0.0461, R_{sigma} = 0.0453]	2974 [R_{int} = 0.0237, R_{sigma} = 0.0243]
Data/restraints/parameters	2804/0/258	2974/0/192
Goodness-of-fit on F^2	1.057	1.096
Final R indexes [I>=2σ (I)]	R_1 = 0.0549, wR_2 = 0.1460	R_1 = 0.0442, wR_2 = 0.1233
Final R indexes [all data]	R_1 = 0.0583, wR_2 = 0.1521	R_1 = 0.0473, wR_2 = 0.1257
Largest diff. peak/hole / e $Å^{-3}$	0.73/−0.79	0.33/−0.29

4. References

(1) White C., Yates A., Maitlis P., Heinekey D. (η5-Pentamethylcyclopentadienyl)Rhodium and -Iridium Compounds. *Inorg. Synth.* 1992, 29, https://doi.org/10.1002/9780470132609.ch53

(2) Fujioka H., Murai K., Ohba Y., Hiramatsu A., Kita Y. A mild and efficient one-pot synthesis of 2-dihydroimidazoles from aldehydes. *Tetrahedron Lett.* **2005**, *46*, 2197–2199. 10.1016/j.tetlet.2005.02.025.

(3) Dar'in D. et al. Diazo Tetramic Acids Provide Access to Natural-Like Spirocyclic Δ α,β - Butenolides through Rh(II)-Catalyzed O–H Insertion/Base-Promoted Cyclization // *J. Org. Chem.* **2024**. *89* (11), 7366–7375.

(4) Dolomanov, O. V.; Bourhis, L. J.; Gildea, R. J.; Howard, J. A. K.; Puschmann, H. OLEX2: A Complete Structure Solution, Refinement and Analysis Program. *J. Appl. Crystallogr.* **2009**, *42* (2), 339–341. https://doi.org/10.1107/S0021889808042726.

(5) Sheldrick, G. M. SHELXT - Integrated Space-Group and Crystal-Structure Determination. *Acta Crystallogr. Sect. A Found. Crystallogr.* **2015**, *71* (1), 3–8. https://doi.org/10.1107/S2053273314026370.

(6) Sheldrick, G. M. Crystal Structure Refinement with SHELXL. *Acta Crystallogr. Sect. C Struct. Chem.* **2015**, *71* (Md), 3–8. https://doi.org/10.1107/S2053229614024218.
